# A flagellum-specific chaperone facilitates assembly of the core type III export apparatus of the bacterial flagellum

**DOI:** 10.1371/journal.pbio.2002267

**Published:** 2017-08-03

**Authors:** Florian D. Fabiani, Thibaud T. Renault, Britta Peters, Tobias Dietsche, Eric J. C. Gálvez, Alina Guse, Karen Freier, Emmanuelle Charpentier, Till Strowig, Mirita Franz-Wachtel, Boris Macek, Samuel Wagner, Michael Hensel, Marc Erhardt

**Affiliations:** 1 Junior Research Group Infection Biology of *Salmonella*, Helmholtz Centre for Infection Research, Braunschweig, Germany; 2 Max Planck Institute for Infection Biology, Berlin, Germany; 3 Abteilung Mikrobiologie, Fachbereich Biologie/Chemie, University of Osnabrück, Osnabrück, Germany; 4 Interfaculty Institute of Microbiology and Infection Medicine (IMIT), Section of Cellular and Molecular Microbiology, University of Tübingen, Tübingen, Germany; 5 Junior Research Group Microbial Immune Regulation, Helmholtz Centre for Infection Research, Braunschweig, Germany; 6 Proteome Center Tübingen, University of Tübingen, Tübingen, Germany; 7 German Center for Infection Research (DZIF), Partner-site Tübingen, Tübingen, Germany; UMDNJ/Robert Wood Johnson Medical School, United States of America

## Abstract

Many bacteria move using a complex, self-assembling nanomachine, the bacterial flagellum. Biosynthesis of the flagellum depends on a flagellar-specific type III secretion system (T3SS), a protein export machine homologous to the export machinery of the virulence-associated injectisome. Six cytoplasmic (FliH/I/J/G/M/N) and seven integral-membrane proteins (FlhA/B FliF/O/P/Q/R) form the flagellar basal body and are involved in the transport of flagellar building blocks across the inner membrane in a proton motive force-dependent manner. However, how the large, multi-component transmembrane export gate complex assembles in a coordinated manner remains enigmatic. Specific for most flagellar T3SSs is the presence of FliO, a small bitopic membrane protein with a large cytoplasmic domain. The function of FliO is unknown, but homologs of FliO are found in >80% of all flagellated bacteria. Here, we demonstrate that FliO protects FliP from proteolytic degradation and promotes the formation of a stable FliP–FliR complex required for the assembly of a functional core export apparatus. We further reveal the subcellular localization of FliO by super-resolution microscopy and show that FliO is not part of the assembled flagellar basal body. In summary, our results suggest that FliO functions as a novel, flagellar T3SS-specific chaperone, which facilitates quality control and productive assembly of the core T3SS export machinery.

## Author summary

Many bacteria use the bacterial flagellum for directed movement in various environments. The assembly and function of the bacterial flagellum and the related virulence-associated injectisome relies on protein export via a conserved type III secretion system (T3SS). The multicomponent transmembrane core export apparatus of the flagellar T3SS consists of FlhA/B and FliP/Q/R and must assemble in a highly coordinated manner. In the present study, we determined the role of the transmembrane protein FliO in the maturation of the flagellar core protein export apparatus. We show that FliO functions as a flagellum-specific chaperone during the initial step of export apparatus assembly. FliO facilitates the efficient formation of a stable FliP–FliR core complex and is thus required for quality management and productive assembly of the flagellar export apparatus. Our results suggest a coordinated assembly process of the flagellar core export apparatus that nucleates with the FliO-dependent formation of a FliP–FliR complex. Subsequent incorporation of FliQ, FlhB, and FlhA leads to the assembly of a secretion-competent flagellar T3SS.

## Introduction

The ability of many bacteria to move on surfaces and swim through liquid environments depends on the function of a rotating nanomachine, the bacterial flagellum. The flagellum is highly conserved among bacterial species and is best characterized in *Salmonella enterica* serovar Typhimurium. The structure of the flagellum can be divided into 3 main parts: a basal body, a flexible hook, and a long rigid filament [1,2]. The basal body itself is composed of several substructures located in the cytosol or spanning the bacterial cell envelope. The inner membrane MS ring, the periplasmic P ring, and the outer membrane L ring assemble using the Sec-pathway. The self-assembly of all other axial parts of the flagellum is dependent on protein export through the flagellar-specific type III secretion system (fT3SS) [3]. The core integral-membrane components of the flagellar export apparatus (FlhA/B and FliP/Q/R) are closely related to the virulence-associated T3SS (vT3SS) of the injectisome device used by many gram-negative bacteria to inject toxins into host cells [4]. Protein export via both fT3SS and vT3SS is primarily dependent on the proton motive force (pmf) [5–9]. How the bacterial cell coordinates the self-assembly of the multicomponent export apparatus complex in the inner membrane remains elusive.

Six cytoplasmic and seven membrane proteins are essential for the export of the majority of the extra-cytoplasmic building blocks of the flagellum. The MS ring is made of 26 subunits of FliF and likely assembles after the completion of the core integral membrane export apparatus, similar to the injectisome system [10–13]. Tightly associated with the MS ring is a cytoplasmic ring (C ring) made of FliG/M/N. The C ring functions as a rotor/switch complex and serves also as a docking platform for cargo [14] and the FliH/I/J ATPase complex [15,16], which facilitates export via ATP hydrolysis [7,17]. Within the MS ring, the integral membrane proteins FlhB/A and FliO/P/Q/R are thought to form the export gate. FlhA was reported to form a nonameric ring and presumably energizes export using the pmf [11,18–20]. FlhB is involved in the switch of secretion specificity between late and early substrates [21]. FliO (17.5 kDa), FliP (25 kDa), FliQ (9 kDa), and FliR (26 kDa) are integral membrane proteins and essential for the export of flagellar substrates [22]. FliP/Q/R are highly conserved in all fT3SS and have homologs in the vT3SS [23]. Interestingly, FliO homologs are apparently absent in the vT3SS, suggesting an important fT3SS-specific role [24]. FliO is a bitopic membrane protein with a large cytosolic C-terminal domain. While FliO is required for flagella formation and motility under physiological conditions, upregulation of flagellar secretion substrates and a secondary-site suppressor mutation in FliP restored partial motility of a Δ*fliO* strain, indicating a functional link between FliO and FliP [25–27].

Here, we determined the molecular function of FliO in maturation of the flagellar type III secretion system (T3SS). We propose that FliO functions as a flagellum-specific, integral membrane chaperone that stabilizes FliP protein and facilitates the formation of a stable FliP–FliR core complex, which is essential for the productive assembly of a functional flagellar basal body.

## Results

### Phylogenetic distribution of FliO reveals widespread conservation

Homologs of FliO are absent in vT3SS of the injectisome and were also thought to be absent in many flagellated species. However, Pallen et al. [28] suggested that FliO homologs are misannotated as FliZ in some bacterial species and identified several potential FliO homologs through PSI-BLAST searches: Cj0352 in *Campylobacter jejuni*, LA2612 in *Leptospira interrogans*, RB9276 in *Rhodopirellula baltica*, and TP0719 in *Treponema pallidum*.

**Fig 1:**
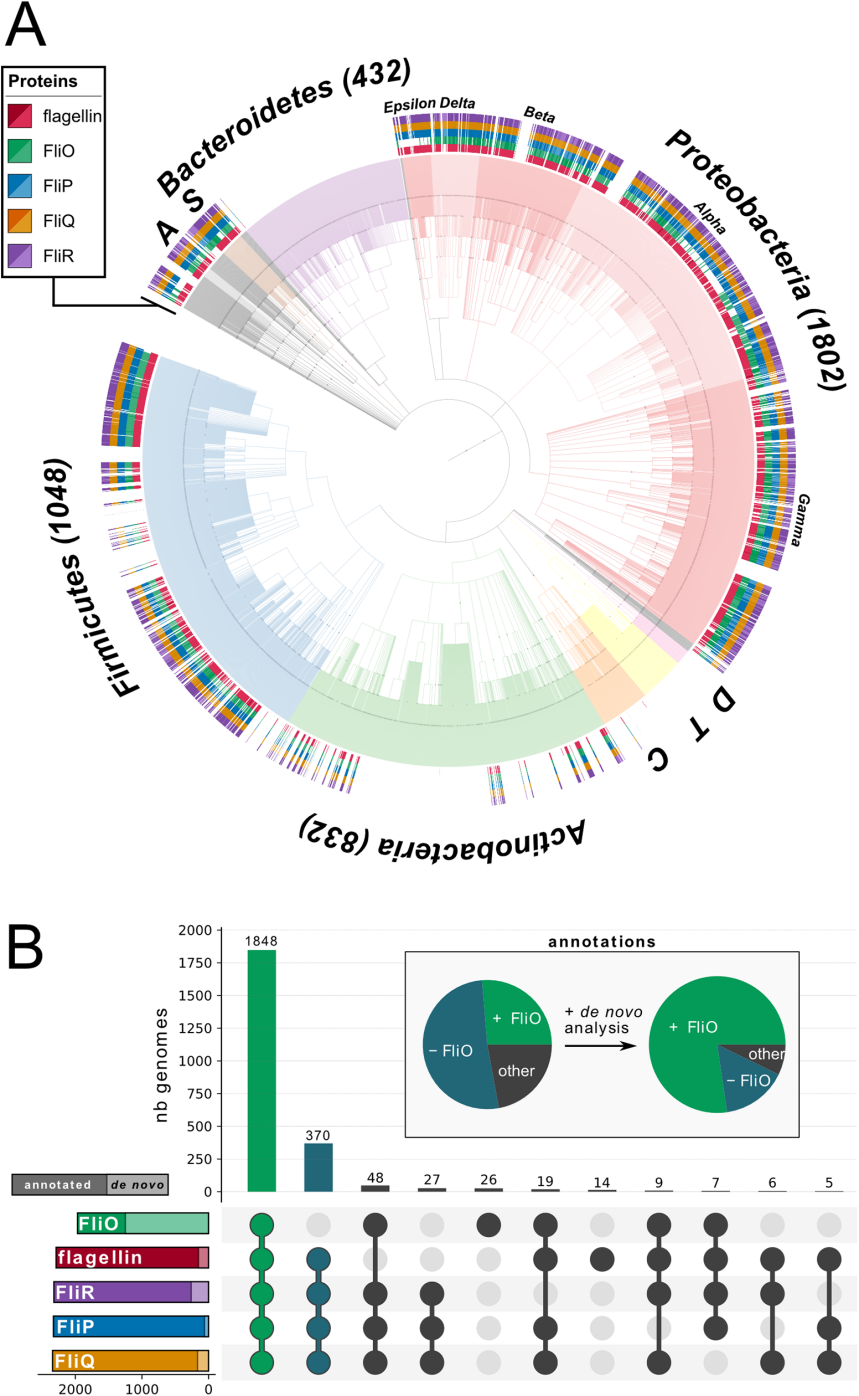
Computational prediction and phylogenetic distribution of FliO, FliP, FliQ, FliR, and flagellin across all bacterial phyla. The distribution of FliO, FliP, FliQ, FliR, and flagellin in all The National Center for Biotechnology Information (NCBI) reference genomes (*n* = 4771) was analyzed according to NCBI annotation and de novo prediction using a Hidden Markov Model (HMM) based on curated Pfam (protein family) database models. (A) Phylogenetic tree based on NCBI taxonomy—outer rings indicate the presence/absence of flagellin, FliO, FliP, FliQ, and FliR. Each colored branch highlights a bacterial phylum. The number of retrieved genomes is indicated for the major phyla; C = Cyanobacteria (119), T = Tenericutes (116), S = Spirochaetes (60), D = Deinococcus-Thermus (39), A = Acidobacteria (24). (B) Gene set representation of de novo predicted FliO, FliP, FliQ, FliR, and flagellin. Left bars show the total number of predicted proteins including previously annotated (dark color) and newly predicted ones (light color) across the NCBI reference genomes. The top bars represent the number of genomes for each combination of predicted FliO, FliP, FliQ, FliR, and flagellin. The pie chart highlights the improved annotation of FliO through the HMM de novo prediction.

This suggested that FliO homologs are more widespread than previously thought, and we thus performed a detailed phylogenetic analysis of the distribution of FliO proteins across different bacterial phyla (Fig 1). We retrieved the full collection of representative genomes from the *refseq* NCBI repository (*n* = 4771) and queried the genomes for the presence of flagellin, FliO, FliP, FliQ, and FliR homologs using regular annotations. We found that FliO homologs in particular were poorly annotated and in fact sometimes misannotated as FliZ, as previously noted by Pallen et al. [28]. This suggested that a large proportion of FliO homologs are missed by the automated annotation algorithms.

We next performed a de novo prediction of flagellin, FliO, FliP, FliQ, and FliR homologs using Hidden Markov Models (HMMs) based on curated Pfam (protein family) database models (Fig 1, S1 Fig). We screened all genomes using the HMM and identified a large number of FliO homologs in genomes in which also FliP and other flagella components were predicted. The majority of predicted FliO hits corresponded to hypothetical proteins, and we identified possible FliO homologs in >80% of flagellated bacteria across most bacterial phyla (Fig 1).

### FliO protects FliP from proteolytic degradation

**Fig 2:**
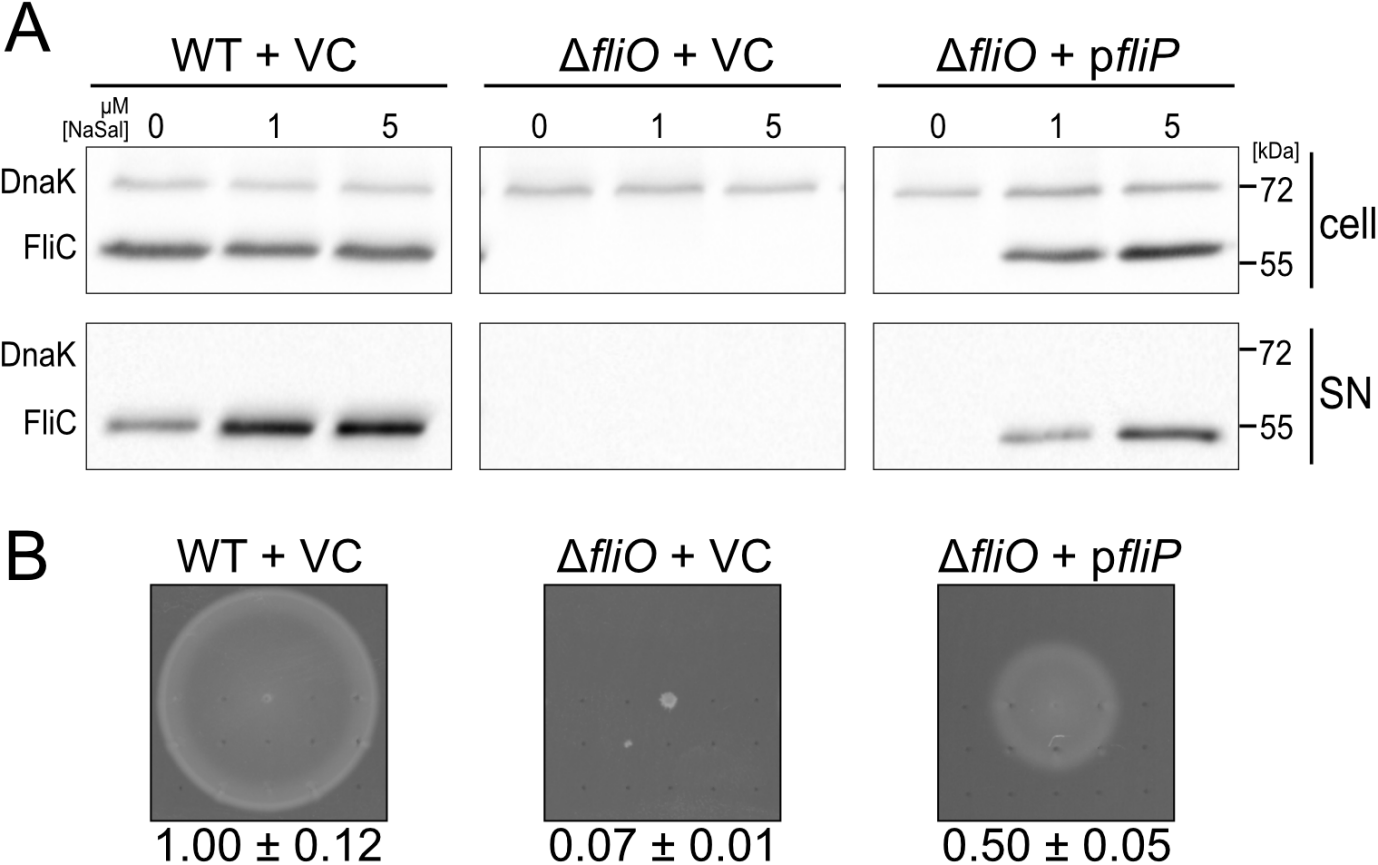
Excess FliP bypasses the requirement of FliO for flagellar-specific type III secretion system (fT3SS) function and motility. (A) Flagellin secretion in the wild-type (WT) (EM2320) and the Δ*fliO* mutant (EM2323, EM2324) harboring an empty (vector control [VC]) or *fliP* (p*fliP*)-expressing vector. Complementation was induced by the addition of 0 μM, 1 μM, and 5 μM sodium salicylate (NaSal). Secreted flagellin was detected by Western blot using anti-FliC antibodies. DnaK was used as loading control. (B) Motility of the WT and the Δ*fliO* mutant complemented with the empty (VC) or *fliP* (p*fliP*)-expressing vector in 0.3% swim agar plates after 5 h at 37°C. Expression of *fliP* was induced using 5 μM NaSal. Relative motility reports the mean ± SD, *n* = 4.

The gene encoding for FliP is frequently encoded in an operon with *fliO*, and a previous study suggested a functional link between FliO and FliP based on genetic evidence [26]. FliP is strongly conserved in both fT3SS and vT3SS (S2 Fig) and essential for protein export [22]. Accordingly, we hypothesized that the flagellum-specific protein FliO evolved to facilitate efficient production or assembly of FliP, a core component of the fT3SS. In an attempt to validate the presumed functional link between FliO and FliP, we analyzed the effect of excess FliP and FliO in wild-type (WT) and Δ*fliO* backgrounds. In the WT background, neither expression of FliP nor FliO increased motility compared to the empty vector control (VC) (Fig 2 + S3 Fig). In the *fliO* deletion background, overproduction of FliP but not of any other fT3SS integral membrane protein restored motility (S4 Fig). These results confirmed that FliO is functionally related to FliP.

The dispensability of FliO upon FliP overexpression led us to speculate that FliO was involved in maintaining FliP stability. To follow this idea, we monitored FliP protein levels after the arrest of de novo protein synthesis. We replaced the native *fliP* gene with epitope-tagged FliP variants to facilitate the detection of FliP protein. The 3×hemagglutinin (HA)- or 3×FLAG-tagged FliP proteins were expressed from the physiological *fliP* locus and retained WT function in a swimming plate motility assay (S5 Fig).

**Fig 3:**
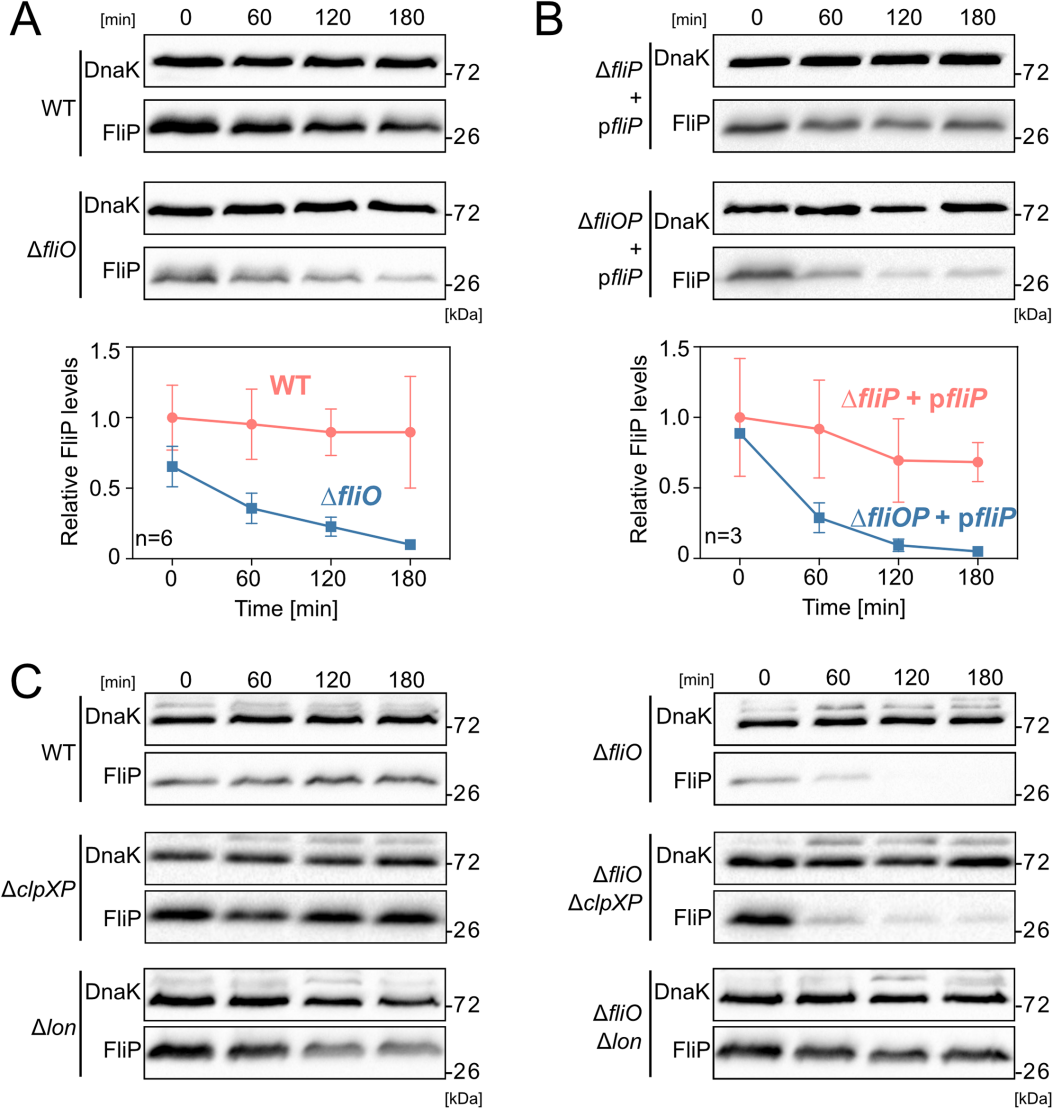
FliP protein is unstable in the absence of FliO. (A) FliP protein stability in the presence and absence of FliO. Protein levels of chromosomally expressed FliP-3×FLAG were monitored at 0, 60, 120, and 180 min after arrest of de novo protein synthesis. Wild-type (WT) (EM2225), Δ*fliO* (EM3201). FliP protein levels were normalized to DnaK, and relative FliP levels report the mean ± SD, *n* = 6. (B) Stability of episomally expressed FliP-3×HA protein in Δ*fliP* and Δ*fliOP* mutants after arrest of de novo protein synthesis. Δ*fliP* + p*fliP* (TH17448), Δ*fliOP* + p*fliP* (EM1610). Relative FliP levels report the mean ± SD, *n* = 3. (C) Protein stability of chromosomally expressed FliP-3×HA in presence or absence of FliO in the WT (TH17323), Δ*fliO* (EM1274), Δ*clpXP* (EM4018), Δ*clpXP* Δ*fliO* (EM4019), Δ*lon* (EM4478), and Δ*lon* Δ*fliO* (EM4479) mutants.

In a WT background, FliP remained stable after synthesis inhibition during the duration of the experiment. In a Δ*fliO* mutant, however, FliP protein levels degraded rapidly (Fig 3A). This suggested that FliO was involved in stabilizing FliP protein. To exclude polar effects of the *fliO* deletion, we monitored protein stability of episomally expressed FliP in Δ*fliP* and Δ*fliOP* mutant backgrounds and observed a similar degradation pattern of FliP in the absence of FliO (Fig 3B). Reminiscent of the FliP degradation pattern in a Δ*fliO* mutant, we observed rapid degradation of FliP in nonfunctional FliO point mutants (V72G, L91R, and ΔL91-L94) (S6 Fig). Similar FliO point mutations (L91A, ΔL91) were previously shown to be expressed but not able to complement a *fliO* null mutant [26].

As mentioned above, FliP harbors a signal peptide of the Sec-system, and this raised the possibility that FliO participated in efficient cotranslational membrane integration of FliP. We thus assessed the effects of a *fliO* deletion in respect to the level of membrane-integrated FliP. Crude membranes were collected by ultracentrifugation and washed with urea to discriminate properly integrated proteins from aggregated proteins associated with the cytoplasmic membrane. FliP protein levels were determined in both the presence and absence of FliO (S7 Fig). As expected for a polytopic integral membrane protein, we detected FliP only in the membrane fractions but not in the cytosol. FliP steady-state protein levels were comparable in the presence and absence of FliO, indicating that FliO did not influence FliP incorporation into the inner membrane.

In order to understand the mechanisms underlying FliO stabilization of FliP, we aimed to identify the protease responsible for FliP degradation. We first analyzed the contribution of the integral membrane protease FtsH, which is involved in quality control of membrane protein complexes, such as the SecYEG translocase [29]. As a deletion of FtsH is lethal, we monitored FliP protein stability under conditions overproducing an inhibitor of FtsH, STM1085 (YccA in *Escherichia coli*) [30,31]. YccA has been shown to limit the suicidal degradation of jammed Sec-translocon complexes by inhibiting FtsH activity [31]. LamB-LacZ hybrids are commonly used to study jammed Sec-translocons. In order to verify the functionality of our YccA expression construct, we thus monitored bacterial growth after jamming of the Sec-translocon using a LamB-LacZ hybrid in the presence or absence of excess YccA. Expression of YccA from a medium-copy plasmid rescued the lethal growth defect after induction of the LamB-LacZ hybrid (S8A Fig). We next analyzed FliP protein stability in the absence of FliO in the presence or absence of YccA, the inhibitor of FtsH. As shown in S8B Fig, the FliP degradation pattern did not change, which suggested that FtsH might not be primarily responsible for the observed proteolytic degradation of FliP in the absence of FliO. We therefore analyzed FliP stability in the absence of FliO in mutants defective for the cytoplasmic ClpP and Lon proteases. In the Δ*clpXP* mutant, the presence of FliO was required to maintain FliP stability. In the Δ*lon* mutant, however, FliP was also significantly more stable in the absence of FliO, suggesting that Lon is the primary protease responsible for FliP degradation (Fig 3C).

### FliO is not located in the basal body of the flagellum

**Fig 4:**
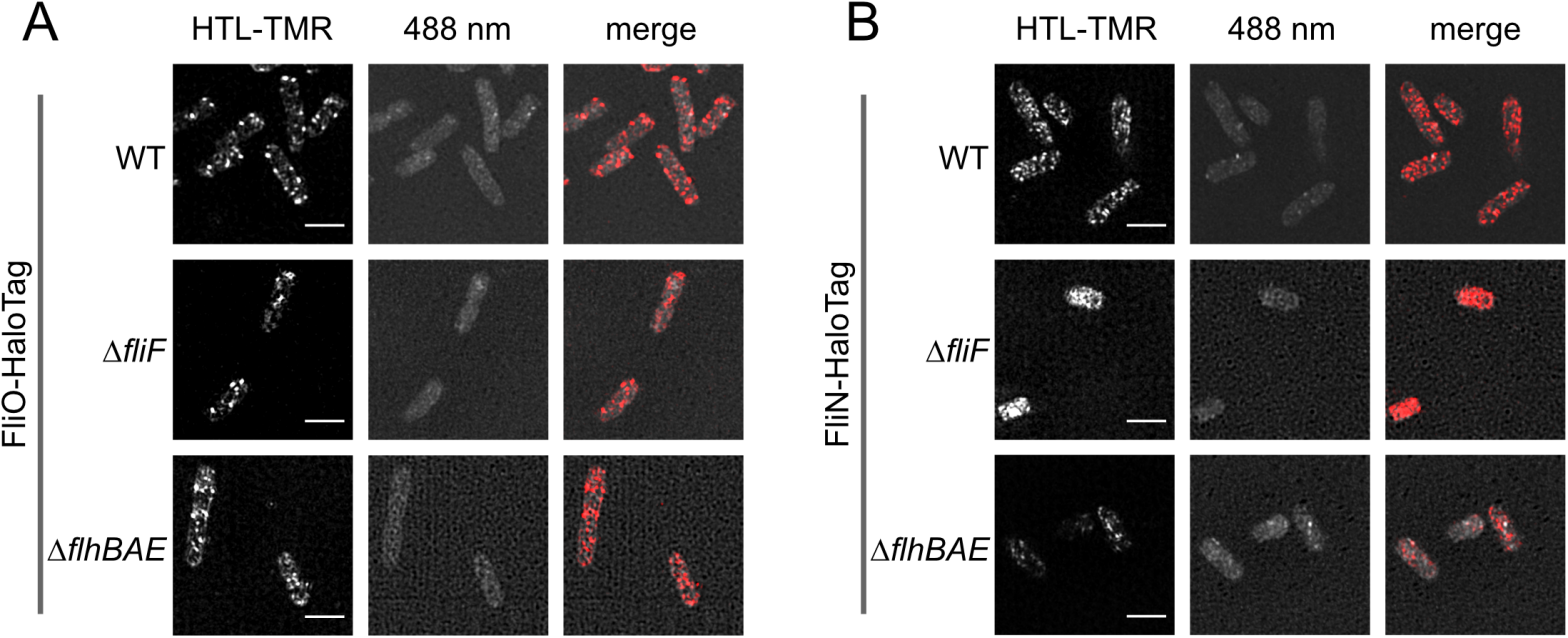
Subcellular localization of FliO revealed by structured illumination microscopy. The subcellular localization of FliO-HaloTag (A) or FliN-HaloTag (B) fusions expressed from their native chromosomal locus was analyzed in the wild-type (WT) and mutant backgrounds defective in MS-ring assembly (Δ*fliF*) or flagellar-specific type III secretion system (fT3SS) function (Δ*flhBAE*). WT FliO-HaloTag (EM1077), WT FliN-HaloTag (EM1081), Δ*fliF* FliO-HaloTag (EM6254), Δ*fliF* FliN-HaloTag (EM2640), Δ*flhBAE* FliO-HaloTag (EM6256), and Δ*flhBAE* FliN-HaloTag (EM6258). Strains were treated with 20 nM HaloTag ligands (HTL tetramethylrhodamine [TMR]) and observed using structured illumination microscopy (SIM). The autofluorescence of bacteria upon excitation with a 488 nm laser is shown in the middle panels. Scale bar 2 μm.

**Fig 5:**
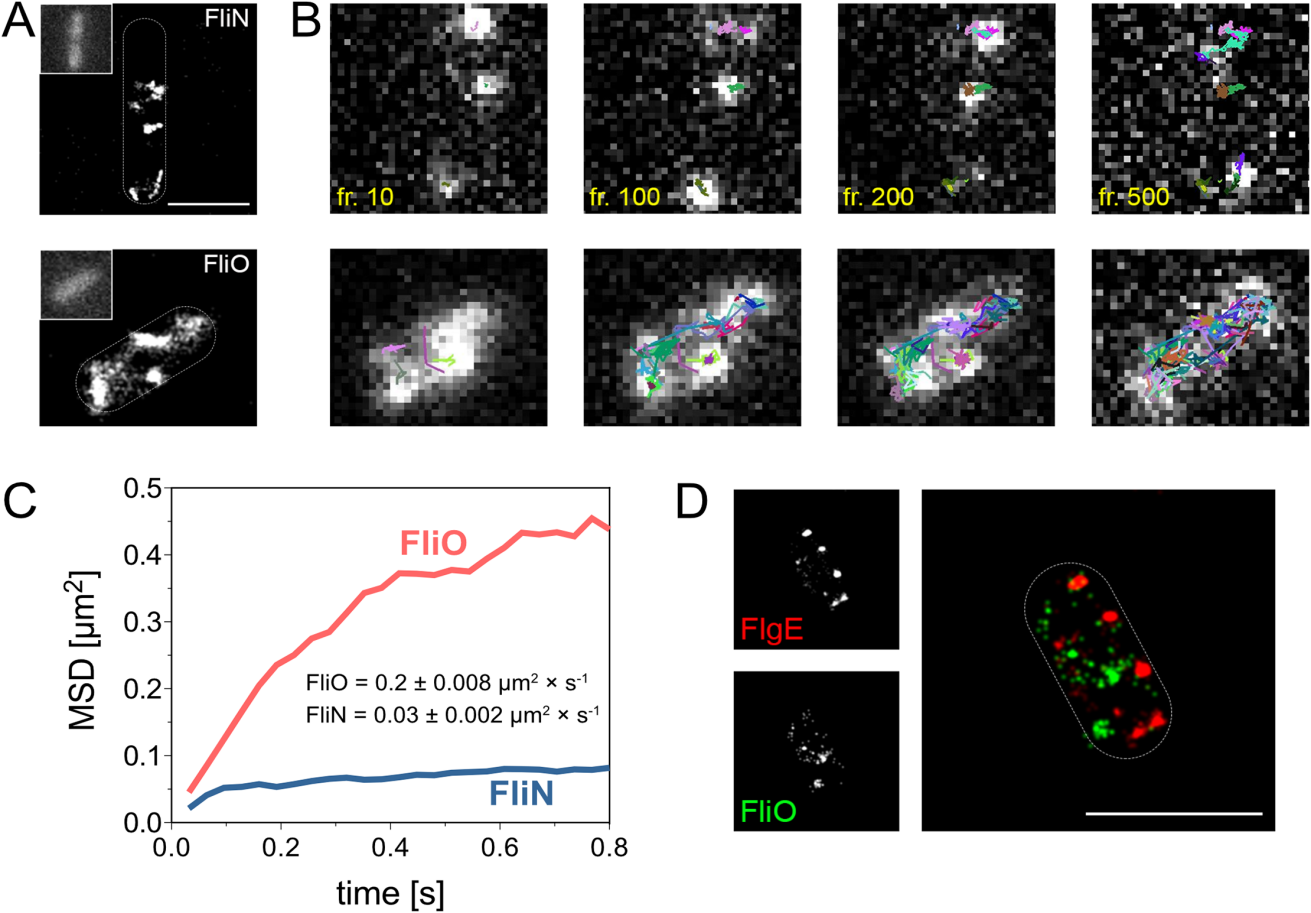
Single-particle tracking of FliO and colocalization with the flagellar basal body. (A) Strains expressing chromosomal FliN-HaloTag (EM1081) or FliO-HaloTag (EM1077) fusions were treated with 20 nM HaloTag ligands (HTL tetramethylrhodamine [TMR]) and analyzed by total internal reflection fluorescence (TIRF) microscopy. As described before, 500 frames were acquired with 5 mW laser power at the focal plane. The autofluorescence of bacteria upon excitation with a 488 nm laser is shown in the upper left corner. Scale bar 1 μm. (B) Single-molecule tracking (SMT) of TMR-labeled FliN and FliO. Selected frames from a series of 500 frames are shown, and frame numbers are indicated. (C) Mean square displacement (MSD) plots of pooled trajectories of at least 25 bacteria recorded under the same conditions. The diffusion coefficient *D* was calculated using the Jaqaman algorithm. (D) Dual-color direct stochastic optical reconstruction microscopy (dSTORM) of fixed bacteria expressing chromosomal FlgE-3×HA and FliO-HaloTag fusions (EM1214). Scale bar 1 μm.

Previous studies suggested that FliO, FliP, FliQ, and FliR were integral parts of the basal body complex [22,32]. However, the subcellular localization of the fT3SS components has only been experimentally determined for FliP and FliR [23]. As we demonstrated above, overproduction of FliP readily bypassed the requirement of FliO for the formation of functional flagella, which suggested that FliO might not be part of the completed fT3SS. To determine the subcellular localization of FliO, we performed single-molecule super-resolution microscopy of FliO-HaloTag and FliN-HaloTag protein fusions using structured illumination microscopy (SIM) and direct stochastic optical reconstruction microscopy (dSTORM) [27,33]. The chromosomal HaloTag fusions to FliO and FliN did not impair the protein export function of the fT3SS (S9 Fig), and the FliN-HaloTag construct has previously been shown to display normal flagellation patterns [27]. The C ring protein FliN was localized in clusters corresponding to the C rings of flagellar basal bodies, as previously reported [14,34], but not in the absence of the MS ring needed for C ring assembly (Fig 4, Fig 5A). FliO, however, was evenly distributed in smaller clusters in the cytoplasmic membrane, and the observed FliO-HaloTag clusters were not affected in mutant strains defective in MS-ring or fT3SS assembly (Fig 4, Fig 5A). Single-molecule tracking (SMT) revealed that FliN remained stably attached in the cell envelope with an apparent diffusion coefficient of 0.03 μm^2^ s^−1^. FliO followed long trajectories in the cell envelope with a 6-fold higher apparent diffusion coefficient of 0.2 μm^2^∙s^−1^ (Fig 5B and 5C), which is consistent with previous reports ranging from 0.02 to 0.2 μm^2^∙s^−1^ for freely diffusing inner-membrane proteins [35,36]. We next performed colocalization studies of FliO-HaloTag and the C ring protein FliM-mEos (S10 Fig) and FliO-HaloTag or FliN-HaloTag with the flagellar hook (S10 Fig, Fig 5D). The hook-basal-body (HBB) components FliM, FliN, and epitope-tagged hook protein FlgE localized in discrete spots similar to FliN-HaloTag as described above, and, consistently, FliN-HaloTag complexes were readily identified in the vicinity of extracellular hook structures (S10 Fig). In contrast, we did not observe colocalization of FliO-HaloTag with HBB components (S10 Fig, Fig 5D). These results indicated that FliO is not part of the final assembled fT3SS within the basal body complex. We thus hypothesized that FliO does not actively participate in the protein export process but acts as an accessory protein during the assembly of the core export apparatus.

### FliO is required for FliP complex formation and basal body assembly

**Fig 6:**
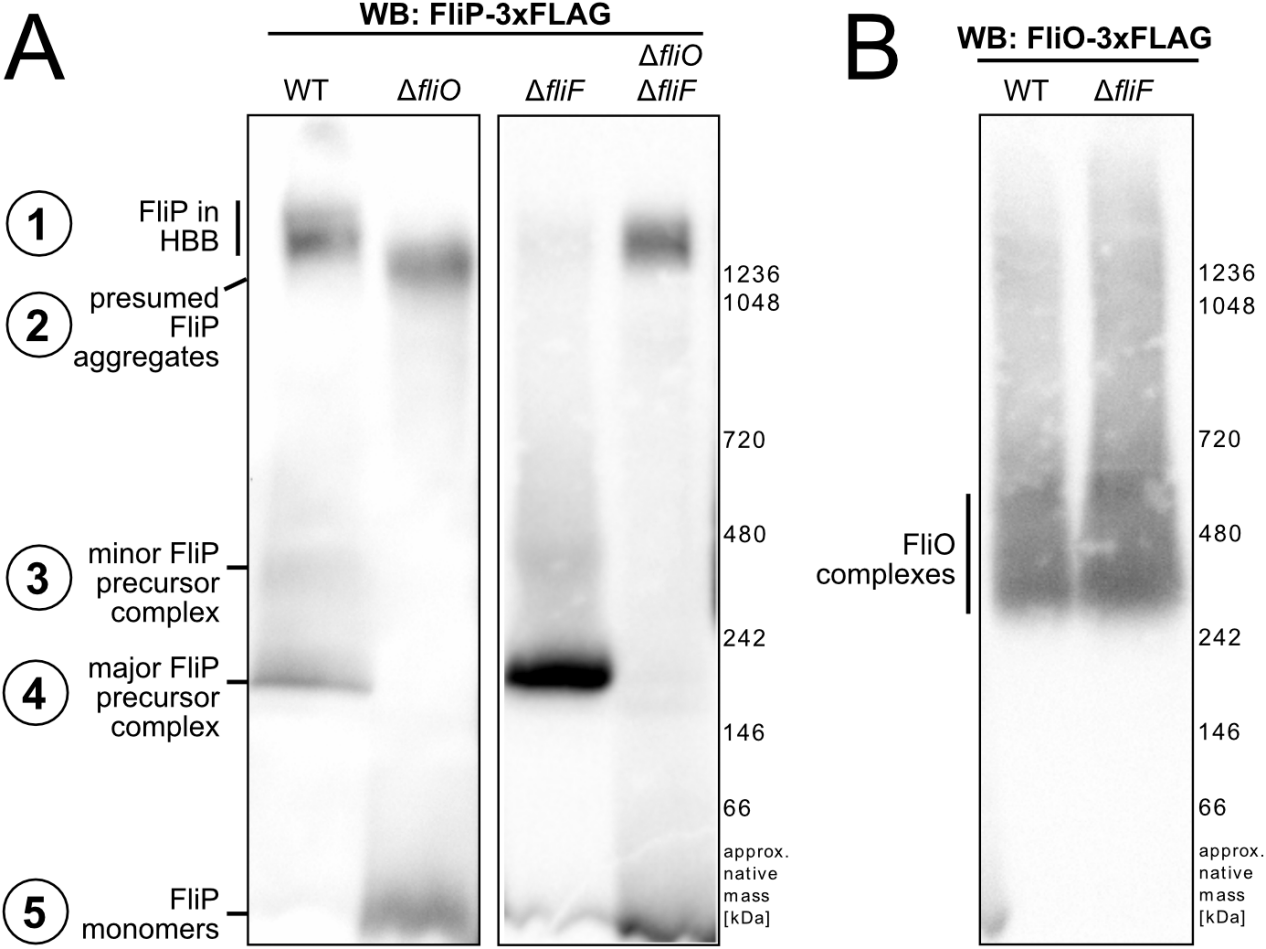
FliP subcomplex formation revealed by blue native PAGE (BN-PAGE). A. Anti-FLAG Western blot of BN-PAGE of crude membrane extracts prepared from the wild-type (WT) (EM2225), Δ*fliO* (EM3201), Δ*fliF* (EM4909), and Δ*fliO* Δ*fliF* (EM4910) mutant strains encoding for chromosomal FliP-3×FLAG. (B) Anti-FLAG Western blot of BN-PAGE of crude membrane extracts prepared from the WT (EM2269) and Δ*fliF* (EM3910) mutant strains encoding for chromosomal FliO-3×FLAG.

In order to study the effect of the absence of FliO on core fT3SS export apparatus formation and basal body assembly, we performed blue native PAGE (BN-PAGE) on crude membrane extracts solubilized in n-dodecyl-β-D-maltoside (DDM) (Fig 6). In a WT background, we detected 3×FLAG epitope–tagged FliP expressed from its native chromosomal locus in several low, intermediate, and high-molecular-weight complexes. We additionally performed liquid chromatography-tandem mass spectrometry (LC-MS/MS) analysis of selected bands corresponding to FliP-containing complexes, as detected by BN-PAGE Western blotting (S11 Fig). According to the BN-PAGE migration pattern and mass spectrometry analysis, FliP is present in several complexes in the WT: in a high-molecular-weight complex (complex 1), which presumably corresponds to finished, completed HBB complexes; in a minor (less abundant), intermediate molecular weight precursor complex (complex 3); and in a major (more abundant), intermediate molecular weight precursor complex (complex 4) (Fig 6A). LC-MS/MS analysis of the intermediate-molecular-weight FliP-containing complexes 3 and 4 further suggested the presence of FliO (in complex 3 and 4) and FliR (in complex 4). We note that we did not detect FliP in complex 4 by LC-MS/MS analysis, which is likely explained by well-known difficulties in detecting hydrophobic membrane proteins by LC-MS/MS (S11A Fig).

In a mutant strain deficient for FliO, however, we neither detected FliP in complex 1 corresponding to the completed HBB, nor at the molecular weight of the intermediate-molecular-weight FliP precursor complexes 3 and 4. However, monomeric FliP was substantially more abundant in the Δ*fliO* mutant than in the WT. We also observed the formation of another FliP-containing high-molecular-weight complex in the Δ*fliO* mutant, which apparently migrated slightly below the completed basal body complexes of the WT and could correspond to aggregated FliP subassemblies (complex 2) (Fig 6A).

We next analyzed the assembly of FliP complexes in a mutant strain defective in MS-ring assembly (Δ*fliF*). Interestingly, the formation of the intermediate-molecular-weight FliP-containing subcomplexes 3 and 4 was not dependent on the presence of the MS ring, in contrast to the presence of FliP in the high-molecular-weight basal body complex (complex 1) (Fig 6A). Importantly, in a Δ*fliO* Δ*fliF* double mutant, we observed the formation of a FliP-containing aggregation complex (complex 2) but not the intermediate-molecular-weight FliP precursor complexes 3 and 4. This suggested that complex 2 corresponds to aggregated FliP and demonstrates that the complex 2 is not related to the completed basal body complex observed in the WT (complex 1). In summary, these results suggested a sequence of assembly of the flagellar core export apparatus, which nucleates with the formation of a FliP-containing subcomplex, followed by subsequent assembly of additional transmembrane export apparatus components, and concluded by the assembly of the MS ring. Similar to the assembly of FliP, the formation of a FlhA-containing high-molecular-weight complex, which presumably corresponds to the finished HBB, was dependent on the presence of FliO and FliF (S12 Fig).

We next performed BN-PAGE of crude membrane extracts of a strain encoding for a functional FliO–FLAG fusion protein to corroborate the mass spectrometry and subcellular localization results. FliO showed a very heterogeneous size distribution in the BN-PAGE analysis, indicating complexes of different compositions (Fig 6B). The presence of FliO in intermediate-molecular-weight FliP-containing complexes—but not in completed HBB complexes—was also supported by LC-MS/MS analysis (S11A Fig) and is consistent with the super-resolution subcellular localization studies of FliO-HaloTag described above.

To validate the presence of FliO in FliP precursor complexes, we performed a FLAG-pull down of chromosomally encoded FliP-3×FLAG in a rod^−^ background (Δ*flgBC*) under native conditions, followed by BN-PAGE protein separation and LC-MS/MS analysis (S11B Fig, S13A Fig). A rod^−^ background was used to facilitate access of the 3×FLAG–tag located in the periplasmic domain of FliP. We observed several high-molecular-weight complexes, which corresponded to different FliP-containing subassemblies of the export gate and the basal body. Mass spectrometry analysis of the FliP-containing complexes suggested the presence of basal body components (FliF, FliG, FlhA) in the high-molecular-weight complexes A and B, and of FlhA, FliO, and FliR in the intermediate-molecular-weight complexes C and D that copurified with FliP. In support, a pull down of chromosomally encoded FliO-3×FLAG using crude membrane extracts of a rod^−^ background copurified chromosomally encoded FliP-3×HA (S13B Fig). We further expressed a plasmid-based FliO His_6_-FliP construct in *E*. *coli* and performed His_6_-FliP affinity purification followed by size exclusion chromatography. High molecular weight, pore-like FliP-containing complexes copurified with FliO, suggesting a direct or indirect interaction between these proteins (S13C Fig).

**Fig 7:**
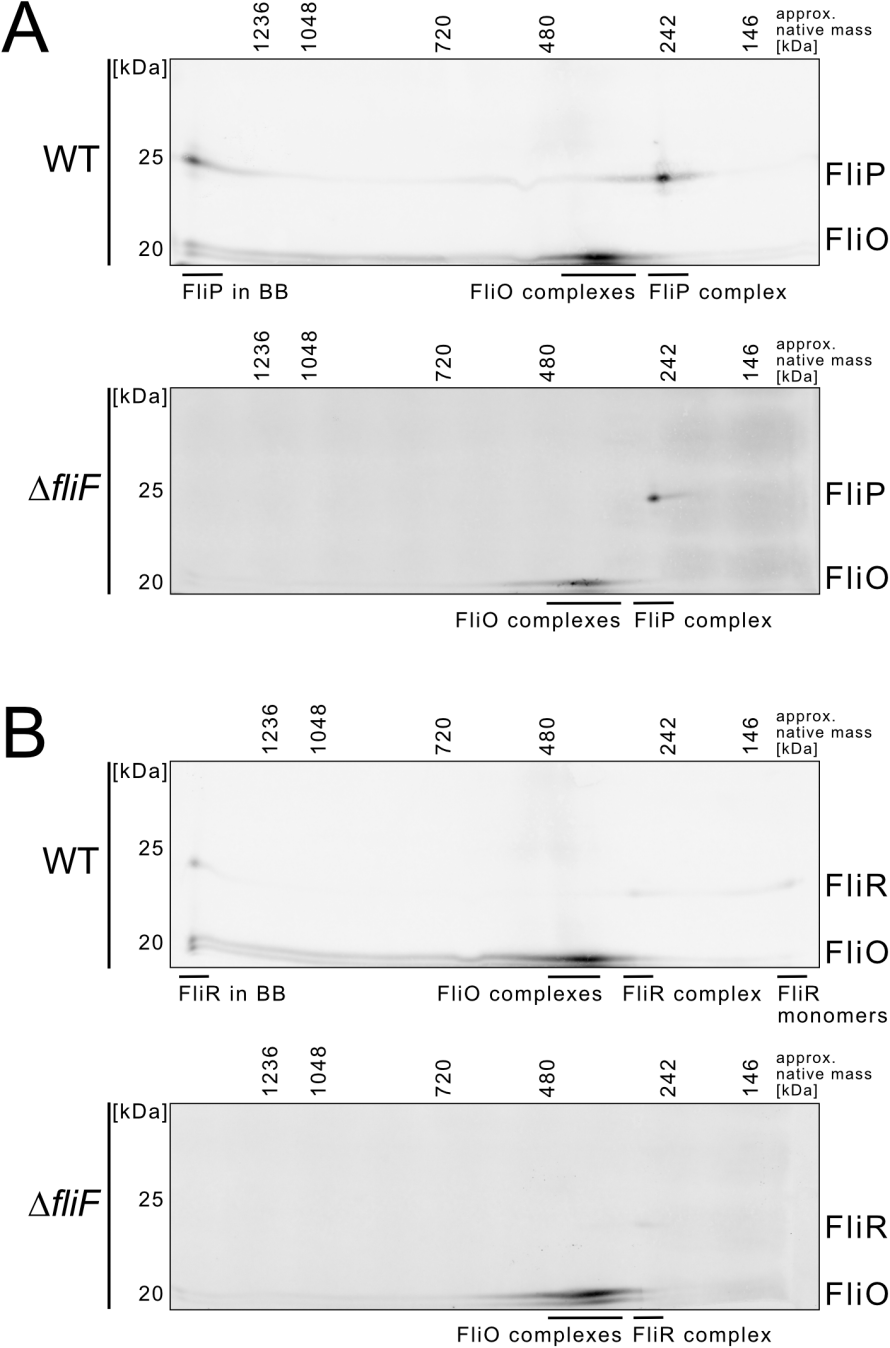
Evidence for FliP–FliR complex formation revealed by 2D blue native PAGE (BN-PAGE). (A) Anti-FLAG Western blot of a 2D BN-PAGE of crude membrane extracts prepared from rod^−^ (Δ*flgBC*) strains encoding for chromosomal FliO-3×FLAG and FliP-3×FLAG. The strains additionally harbored a deletion of *rflP*, a negative regulator of the flagellar master regulator FlhDC and responsible for the phenotypic heterogeneity of flagellar gene expression in lysogeny broth (LB) medium [38]. The deletion of *rflP* results in homogeneous flagella production for cultures grown in LB and thus facilitates detection of the chromosomally encoded epitope-tagged transmembrane export apparatus components, which are expressed at low levels. Wild-type (WT) (EM6229) and Δ*fliF* (EM6195). (B) Anti-FLAG Western blot of a 2D BN-PAGE of crude membrane extracts prepared from Δ*flgBC* Δ*rflP* strains encoding for chromosomal FliO-3×FLAG and FliR-3×FLAG. WT (EM6228) and Δ*fliF* (EM6196).

We next followed up on the presumed presence of the core integral membrane fT3SS component FliR in FliP-containing intermediate-molecular-weight complexes. We purified crude membrane extracts of rod^−^ strains expressing FliP-3×FLAG and FliR-3×FLAG constructs from their native chromosomal locus and performed 2D BN-PAGE analysis (Fig 7). In the WT, we detected FliP and FliR in a high-molecular-weight complex corresponding to the flagellar basal body and in similar intermediate-molecular-weight complexes, which presumably corresponded to FliP–FliR subassemblies. Consistent with the 1D BN-PAGE results of Fig 6, the formation of FliP- and FliR-containing basal body complexes, but not the assembly of the intermediate-molecular-weight FliP–FliR complex (complex 4, Fig 6A), was dependent on the MS-ring protein FliF. We also note that the formation of stable FliP–FliR subassemblies is reminiscent to the previously observed SpaP–SpaR complex of the vT3SS [13,37].

**Fig 8:**
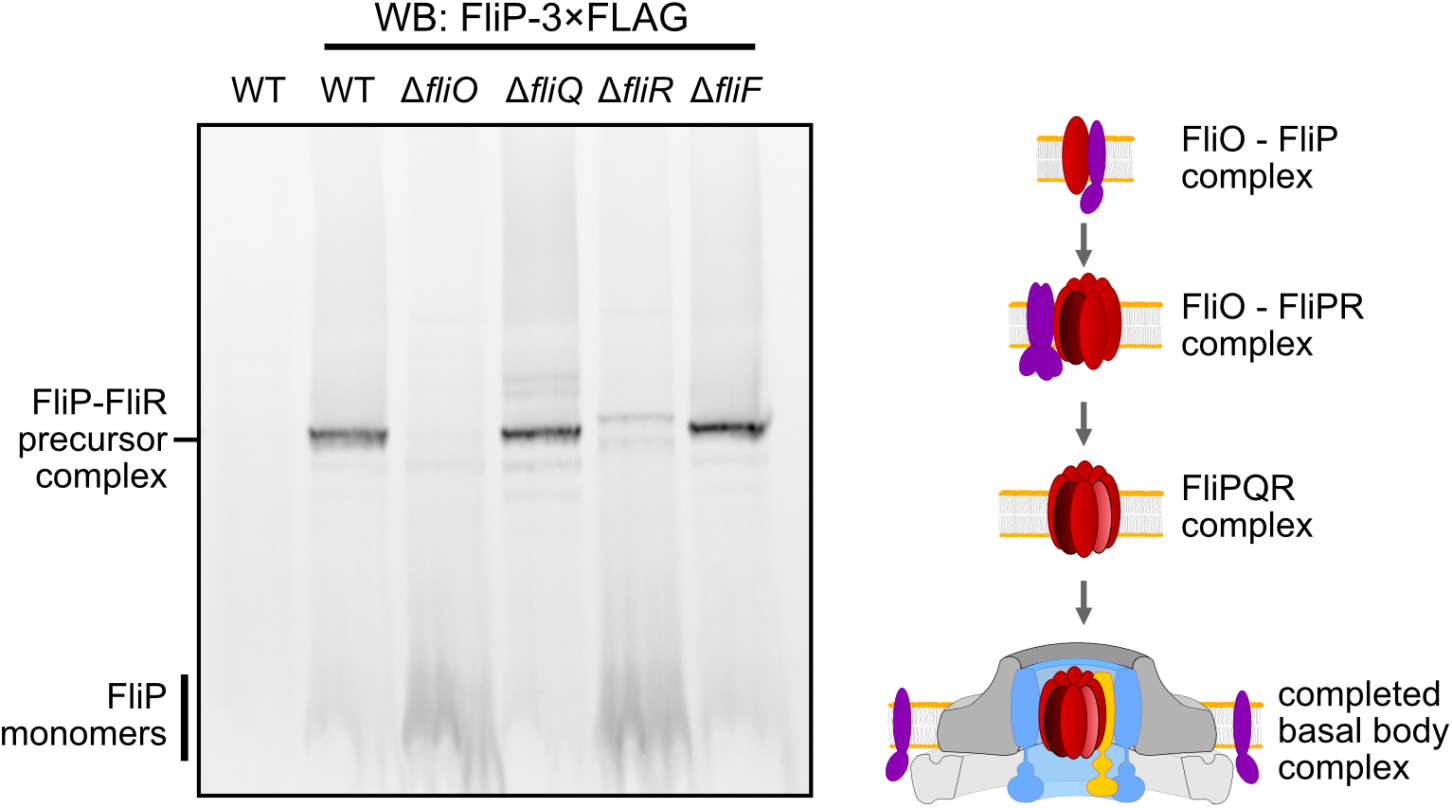
Assembly of FliP subassemblies in core export apparatus mutants and model of the coordinated assembly of the flagellar-specific type III secretion system (fT3SS). Left: The assembly of stable FliP subassemblies is dependent on FliO and FliR but not on FliQ or FliF. Anti-FLAG Western blot of blue native PAGE (BN-PAGE) of crude membrane extracts prepared from the wild-type (WT) harboring untagged FliP (LT2, TH437) and mutant strains encoding for chromosomal FliP-3×FLAG: WT (EM6221), Δ*fliO* (EM6222), Δ*fliQ* (EM6223), Δ*fliR* (EM6224), Δ*fliF* (EM4859). Strains EM6221, EM6222, EM6223, and EM6224 additionally harbored a deletion of the proximal rod components *flgBC* in order to arrest flagellar synthesis after assembly of the core export apparatus. Right: Model of the coordinated assembly of the core flagellar export apparatus. Upon initiation of flagellum assembly, the flagellar type III secretion system (T3SS)-specific chaperone FliO facilitates formation of an oligomeric complex containing FliP and FliR. FliO then presumably dissociates from the stable FliP–FliR core complex. The FliP–FliR core complex forms the nucleus for the assembly of FliQ, FlhB, and FlhA [11], followed by MS-ring (FliF) polymerization and formation of the completed protein export-competent flagellar T3SS.

Our results, presented above, suggested that the observed major, intermediate-molecular-weight FliP precursor complex (complex 4, Fig 6A) contained FliR and that the formation of a stable FliP–FliR complex was dependent on FliO. To test whether formation of the major FliP-containing precursor complex also involves FliQ, whose gene is encoded between *fliP* and *fliR*, we next analyzed formation of the major FliP precursor complex in mutants of the core fT3SS. As shown in Fig 8, the assembly of stable FliP-containing subassemblies was dependent on the presence of FliO and FliR but not on FliQ or FliF. These observations corroborate the hypothesis that FliO facilitates the assembly of a stable FliP–FliR intermediate-molecular-weight complex, which subsequently recruits FliQ and other components of the fT3SS for the assembly of a functional protein export apparatus.

## Discussion

In *Salmonella* and other gram-negative bacteria, the flagellum and virulence-associated injectisome complexes play a crucial role during the infection process. The injectisome is believed to have evolved from the flagellum [39], and both nanomachines share many structural and functional similarities. In particular, the proteins forming the core T3SS protein export apparatus are highly conserved. One of the most striking differences is the absence of a FliO homolog in the injectisome vT3SS. Assembly of the flagellum and bacterial motility is dramatically impaired in the absence of FliO [26]; however, the molecular function of FliO remained elusive. Mutations in *fliP* have been shown to partially restore motility of a Δ*fliO* strain, and overexpression of FliO increased FliP expression, suggesting a functional relationship between these 2 proteins [26]. Here, we show that the nonmotile phenotype of a *fliO* deletion mutant was readily bypassed by moderate overproduction of FliP, which indicated that FliO was involved in FliP stability or complex assembly. To test this hypothesis, we monitored FliP protein stability after arrest of de novo protein synthesis and showed that the presence of FliO prevented FliP degradation. We demonstrate that Lon is the primary protease responsible for FliP degradation. The degradation of FliP as an integral membrane protein by a cytosolic protease is surprising, but LonB of *B*. *subtilis* is membrane-localized, and in yeast, the respective Lon homolog has been shown to degrade proteins of the mitochondrial membrane [40,41]. This result suggests that Lon is able to tightly interact with the cytoplasmic membrane in order degrade misassembled FliP.

It has been thought that FliO was part of the fT3SS within the basal body complex [22,23,32], and we performed super-resolution microscopy analyses to determine the subcellular localization of FliO. FliO did not colocalize with components of the basal body, and single-molecule analysis revealed that FliO complexes are evenly distributed and freely diffusing in the cytoplasmic membrane. We thus hypothesized that FliO is not part of the fT3SS in the assembled basal body and does not actively participate in the export process but rather has a function as an accessory protein during assembly of the core export apparatus.

We tested this hypothesis by performing 1D and 2D BN-PAGE and LC-MS/MS analyses of crude membrane extracts to determine the composition of the basal body complex under native conditions. We did not detect FliO in completed basal body complexes, but FliO was associated with FliP and FliR-containing subcomplexes. The formation of stable FliP–FliR subassemblies required the presence of FliO, but not the MS-ring protein FliF. These observations indicate that precursors of the flagellar export gate assemble prior and independently from the MS ring, as previously shown for vT3SS assembly of the injectisome [37,42]. Finally, FliP monomers detected using BN-PAGE analysis were strikingly more abundant in the absence of FliO, which was consistent with the absence of FliP-containing higher-molecular-weight complexes in the *fliO* mutant.

In summary, we propose that FliO functions as a flagellum-specific chaperone required for productive assembly of the core flagellar export apparatus. We suggest that assembly of the flagellar core T3SS initiates with the formation of FliP–FliR oligomers, which is facilitated by FliO. Upon assembly of a stable FliP–FliR core complex, FliO is released and possibly replaced with other export gate components. The FliP–FliR core complex might subsequently serve as platform for the recruitment of FliQ, FlhB, and the FlhA nonamer, resulting in the formation of a secretion-competent export apparatus needed for flagellar assembly (Fig 8). Both the bacterial flagellum and the homologous vT3SS of the injectisome device are important virulence factors of many pathogenic bacteria. Thus, understanding the principles and molecular mechanisms of the assembly of large, multicomponent transmembrane protein complexes might have important implications for the rational design of novel anti-infectives that interfere with the assembly and function of these nanomachines.

## Materials and methods

### Bacterial strains, plasmids, and growth conditions

Bacterial strains and plasmids used in this study are listed in S1 Table. All strains were derivatives of *S*. *enterica* serovar Typhimurium LT2. Cultures were grown in lysogeny broth (LB) at 37°C under continuous shaking to mid-log phase except when stated otherwise.

### Strain constructions

Chromosomal fusions or clean deletions of genes were created using λ-Red recombination either by *tetRA* cassette replacement using pKD46 plasmid [43] or by *aph-*I-SceI cassette replacement using pWRG730. One-step gene inactivation was performed as described by Datsenko and Wanner [44]. Insertion of a chromosomal LacZ-Frt-aminoglycoside phosphotransferase (Kanamycin resistance)-Frt (FKF) through homologous recombination was performed as described [45]. Mutations, fusions, and deletions were transferred between strains using P22 phage transduction. For STM1085 (*yccA*) cloning, the gene was amplified by PCR from genomic DNA and subcloned blunt end into pBR322 before cloning with DraIII and BstEII into the IPTG-inducible expression plasmid pTr99FFA.

### Phylogenetic analysis and de novo prediction of FliO, FliP, FliQ, FliR, and flagellin homologs

In order to characterize the distribution of FliO, FliP, FliQ, FliR, and flagellin proteins across different bacterial phyla, proteomes (.faa files) of representative genomes were downloaded from *refseq* NCBI: https://www.ncbi.nlm.nih.gov/refseq/about/prokaryotes/.

A total of 4,771 genomes (date: 01.05.2017) were retrieved, and the proteomes were queried based on regular NCBI annotations for the presence of FliO, FliP, FliQ, FliR, and flagellin proteins. For the de novo prediction of homologs of FliO, FliP, FliQ, FliR, and flagellin proteins, HMMs were generated using the curated Pfam database models for FliO (PF04347), FliP (PF00813), FliQ (PF01313), FliR (PF01311), and flagellin (PF00669+PF00700), and all representative genomes were screened using the *hmmsearch* function in HMMER3 [46] with the default Pfam database gathering cutoff (GA) per domain. This HMM approach identified a large number of FliO proteins for genomes in which FliP and the other flagella components have been predicted. The majority of novel FliO proteins corresponded to hypothetical genes. Results from the annotated FliO, FliP, FliQ, FliR, and flagellin proteins and the de novo prediction were integrated using custom Python scripts. For the phylogenetic representation, the NCBI taxonomy IDs were extracted using ETEtools [47], and the tree topology was obtained for a given list of TaxIDs. For tree visualization and rendering, iTOL [48] was used, and set comparison was visualized using the UpSet tool [49].

### Motility assay

Equal amounts of an overnight culture were inoculated in 0.3% swim agar plates supplemented with appropriate additives. Plates were incubated at 37°C and scanned after 4 h to 7 h. The sizes of the motility halos were measured using ImageJ.

### Protein secretion assay

Cells were grown in LB at 37°C until OD_600_ reached 0.8−0.9, and 1.9 ml of culture was harvested and cooled down on ice. Cells and supernatant were separated by 3 × 3 min centrifugation at 18,000 × g at 4°C. The pellet and 1.5-ml supernatant of each strain were precipitated with 10% TCA. After normalization to the OD_600_, samples were loaded on a 15% SDS-PAGE and analyzed by Western blot.

### Protein stability assay

Overnight cultures of chromosomally encoded FliP_Q22_3×HA/FliP_G157_3×FLAG were diluted 1:100 into 25 ml of fresh LB and grown at 37°C under shaking until OD_600_ reached 0.6. Protein synthesis was inhibited by the addition of 0.5 mg ml^−1^ spectinomycin and 12.5 μg ml^−1^ chloramphenicol (Cm). The equivalent of 1 ml at OD_600_ 0.6 was collected at 0, 30, 60, 90, 120, and 180 min after synthesis inhibition. Samples were precipitated in 10% TCA, spun down (30 min at 20,000 x g), washed with ice-cold acetone, and resuspended in 40 μl 2 × SDS loading buffer (300 mM Tris-HCl pH 6.8, 4% SDS, 20% glycerol, 25 mM EDTA, 0.04% bromophenol blue, 2% β-mercaptoethanol). 10 μl of each sample was run on 15% SDS-PAGE and analyzed by Western blot. To assess the effect of STM1085, cultures were grown in LB supplemented with ampicillin and with 1 mM IPTG. Synthesis was stopped and samples were collected after 0 min and 180 min, as described above. For plasmid-encoded FliP_Q22-_3×HA, overnight cultures of Δ*fliOP* and Δ*fliP* harboring pKG116-FliP_Q22-_3×HA were diluted 1/100 into 25-ml LB containing 12.5 μg ml^−1^ Cm and 2.5 μM sodium salicylate (NaSal) and grown at 37°C for 2.5 h until OD_600_ 0.6. Protein synthesis was inhibited by the addition of 0.5 mg ml^−1^ spectinomycin [50]. Samples were collected and treated as described above.

### Western blot

Following SDS-PAGE, proteins were transferred onto a nitrocellulose membrane. Directly after transfer, membranes were stripped at 60°C for 20 min to enhance signal as described previously [51]. Blots were then blocked with 5% milk and probed for 1 h with appropriate antibodies: Anti-HA (Sigma-Aldrich; dilution 1:2,000), Anti-FLAG (Sigma-Aldrich; dilution 1:3,000), Anti-DnaK (Abcam; dilution 1:10,000) mouse monoclonal antibodies, rabbit anti-FliC serum (dilution 1:10,000), and rabbit anti-FlhA serum (dilution 1:10,000), respectively. Membranes were then incubated 30 min in Immun-Star Goat anti-mouse HRP and goat anti-HRP secondary antibodies (1:20,000; BioRad). After washing, membranes were developed using Clarity Western ECL (BioRad) and imaged with a ChemiDoc imaging system (BioRad).

### TCE staining

Total protein amounts were detected before transfer with 2,2,2-Trichloroethanol (TCE), as described previously [52]. Gels were imaged with a ChemiDoc imaging system after 1-min activation.

### Crude and washed membrane preparation for cell fractionation assay

Overnight cultures were diluted 1:50 into fresh LB until OD_600_ 0.7–0.8. The equivalent of 8 OD units was harvested at 8,000 × g for 10 min, resuspended in 750 μl Buffer K (50 mM TEA, 250 mM sucrose, 2 mM EDTA, 10 μg ml^−1^ lysozyme, 10 μg ml^−1^ DNase, 1 mM MgCl_2_, complete protease inhibitors), and incubated for 30 min at 4°C. Cells were lysed using glass beads, and lysates were spun at 10,000 × g for 10 min to eliminate nonlysed cells. Supernatants were then spun down at 150,000 × g at 4°C for 50 min. 900 μl of supernatant was collected and precipitated with 10% TCA, washed, and resuspended in 2 × SDS loading buffer (cytosolic fraction). Pellets were resuspended into 100 μl cold PBS. Half of the suspension was directly mixed with 50 μl 2 × SDS-loading buffer (unwashed membrane fraction). The rest was washed with 1 ml of urea solution (50 mM TAE, 1 mM EDTA pH 7.5) for 1 h at room temperature and centrifuged at 120,000 × g for 1.5 h at 23°C. The supernatant was discarded, and the pellet was resuspended in 100 μl of 1 × SDS loading buffer (washed membrane fraction). All fractions were run on SDS-PAGE and analyzed by Western blots.

### Super-resolution microscopy

(i) Sample preparation and microscopy: Bacteria were subcultured from overnight cultures (1:100) in fresh LB medium and grown for 2.5 h at 30°C. After 1 h 45 min of subculture, fluorescent ligands were added, i.e., 20 nM HTL-TMR (Promega) or 150 nM HTL-Atto655 (self-synthesized) for HaloTag fusions. At least 5 washing steps were performed with minimal medium to remove unbound ligand and LB medium by centrifugation at 8,000 × g for 2 min. Bacteria are diluted to approximately OD_600_ 0.5, and 15 μl was added to freshly prepared agarose-coated glass cover slips prepared with 1% agarose. Another glass slide was positioned on the agarose. Total internal reflection fluorescence (TIRF) microscopy was performed using an inverted microscope (IX71, Olympus) equipped with a motorized 4-line TIRF condenser (cell^TIRF, Olympus), a 150× oil immersion TIRF objective (UAPON 150x OTIRF, Olympus), and high-power lasers: 488 nm, 150 mW (LuxX, Omicron, Germany); 561 nm, 150 mW (Jive, Cobolt, Sweden); 640 nm, 140 mW (LuxX, Omicron, Germany). Images were acquired by an electron-multiplying back-illuminated frame transfer CCD camera (iXon Ultra 897, Andor). A fluorescence filter cube containing a polychroic beamsplitter (R405/488/561/647, Semrock), and a quad-band emission/blocking filter (FF01 446/523/600/677, Semrock) was used. For each cell, 500 frames were recorded with an exposure time of 31 ms for 561 nm and 640 nm laser and laser power of 5 mW. For dual-color dSTORM, bacteria were stained as indicated, washed, and fixed with 3% PFA in PBS for 15 min at RT. After fixation, bacteria were washed again 3 times and were then immobilized on PLL-coated cover slides. As redox system 100 mM β-mercaptoethylamine, 4.5 mg × 10^−1^ ml D-glucose, 40 μg × 10^−1^ ml catalase, and 0.5 μg × 10^−1^ ml glucose-oxidase were added in 1-ml PBS. Five hundred frames were recorded with an exposure time of 31 ms for 561 nm and 640 nm laser, with a laser power of 40 mW and 50 mW, respectively, and a cycle time of 67. SIM was performed using a Nikon Eclipse T_i_ N-SIM super-resolution microscope equipped with a 100× SR ApoTIRF objective and 488 nm and 561 nm lasers. Using an ORCA FLASH 4.0 camera, 100-nm z-stack images were acquired, and the SIM stacks were reconstructed using the Nikon NIS-Elements 4.5.1 software on auto settings. The pixel data of individual z-stacks were subsequently projected on a single plane using maximum intensity settings. (ii) Single molecule localization and tracking: Localization of single molecules as well as SMT were carried out by a self-written graphical user interface written in Matlab R2012 (MathWorks). Single-molecule localization is based on the multiple-target tracing algorithm, and SMT was performed using the utrack algorithm (online available: MTT: http://ciml-e12.univ-mrs.fr/App.Net/mtt/, utrack: http://lccb.hms.harvard.edu/doc/u-track-2.1.3.zip).

### Large-scale crude membrane preparation

In order to perform BN-PAGE, crude membranes were prepared on a large scale as described by Zilkenat et al. [53]. Overnight cultures were diluted 1:100 into 1 liter of fresh LB, and cells were grown at 37°C until OD_600_ 0.8−0.9. All steps were performed at 4°C except when stated otherwise. Eight hundred OD units of culture were harvested and centrifuged at 6,000 × g for 15 min. The supernatant was discarded, and the pellet was washed with cold PBS and centrifuged 10 min at 6,000 × g. Cells were resuspended in 15 ml of buffer K (50 mM TEA, 250 mM sucrose, 1 mM EDTA, pH 7.5) and supplemented with cOmplete mini EDTA free protease inhibitors cocktail, 10 μg ml^−1^ DNase, 10 μg ml^−1^ lysozyme, and 1 mM EDTA. After 30-min incubation, cells were lysed using a French Press at 18,000 psi. After lysis, 1-mM MgCl_2_ was added, and lysates were centrifuged at 24,000 × g for 20 min to eliminate cell debris. Crude membranes were pelleted at 200,000 × g for 50 min, resuspended in 500 μl of buffer M (1×), and homogenized in a 1-ml dounce homogenizer. Membranes were then stored on ice before separation on sucrose gradient or pull down.

### 1D and 2D BN-PAGE

Crude membranes or pull-down elations were prepared as described above. An aliquot of solubilized membranes (45 μl) were collected and mixed to 5 μl of 5% coomassie G (Serva) in 250-mM n-amino-caproic-acid, 50% glycerol and 25 μl were loaded on a 3%–12% NativePAGE (Thermo Fisher scientific) and run for 50 min at 130 V. The cathode buffer was exchanged and the gel was run for an additional 1 h 30 min at 250 V at 4°C. Electrotransfer onto a PVDF membrane was performed at 30 V for 2 h. As previously described [13], 2D BN/SDS PAGE of crude membranes was carried out.

### Sucrose gradient

Previously homogenized crude membrane extracts were carefully deposed at the surface of a 30%−55% sucrose gradient and were centrifuged for 14 h at 150,000 g. All steps were performed at 4°C. Eight fractions of 1 ml were collected and diluted 1:3 with buffer M (50 mM TEA, 1 mM EDTA at pH 7.5) and centrifuged at 200,000 × g for 45 min. The supernatant was discarded, and the membranes were resuspended in 200-μl buffer L (50 mM TEA, 250 mM sucrose at pH 7.5). After SDS-PAGE and Western blot analysis, the purest fractions were pooled together. Protein concentration was measured using Bradford protein assay (BioRad), and the concentration of each sample was adjusted to 3.0 mg ml^−1^. Membranes were then solubilized with 1% DDM for 1 h under gentle shaking and centrifuged 20 min at 20,000 × g to eliminate nonsolubilized membranes. The supernatant was collected, and a 25-μl sample was loaded on a 3%–12% BN-PAGE as described below.

### FliO/FliP-3×FLAG pull down

A strain lacking the rod proteins (Δ*flgBC*) was used to ensure that the FLAG tag was accessible from the periplasm. Crude membranes were prepared as described above from a 1-L culture. Membranes were solubilized for 1 h with 0.5% DDM and centrifuged at 20,000 for 20 min to eliminate cell debris. Solubilized membranes were then incubated for 4 h with 25-μl anti-FLAG M2 affinity gel (Sigma) at 4°C in PBS with 0.1% DDM, washed with 4 column volumes, and eluted by addition of FLAG peptide. After analysis on SDS-PAGE, the purest fractions were pulled together and loaded on a BN-PAGE as described above.

### FliP_His6_ affinity purification

Purification of FliP_His6_ expressed from pTrc99a-*fliO-fliP*_*His6*_ was performed by Ni-NTA chromatography as detailed in Fukumura et al. [54].

### Protein in-gel digestion

Coomassie-stained gel bands were excised and in-gel digested using ProteaseMAX Surfactant (Promega). Sample preparation was done according to the instruction manual with the following modifications: digestions were performed overnight using the enzymes trypsin and chymotrypsin (12.5 ng μl^−1^ each in 20 mM ammonium bicarbonate, 0.01% ProteaseMAX Surfactant). Extracted peptides were desalted using C18 StageTips [55] and subjected to LC-MS/MS analysis.

### Mass spectrometry

LC-MS/MS analyses were performed on an Easy nano-LC (Thermo Scientific) coupled to an LTQ Orbitrap XL mass spectrometer (Thermo Scientific) [56]. The peptide mixtures were injected onto the column in HPLC solvent A (0.1% formic acid) at a flow rate of 500 nl min^−1^ and subsequently eluted with a 57-min gradient of 5-33-50-90% HPLC solvent B (80% ACN in 0.1% formic acid). During peptide elution, the flow rate was kept constant at 200 nl min^−1^. The 10 most intense precursor ions were sequentially fragmented in each scan cycle using collision-induced dissociation (CID). In all measurements, sequenced precursor masses were excluded from further selection for 90 s. The target values for MS/MS fragmentation were 5,000 charges and 10^6^ charges for the MS scan.

### Mass spectrometry data processing

The mass spectrometry data were processed with MaxQuant software suite v.1.5.2.8 [57]. The database search was performed using the Andromeda search engine [58], which is a module of the MaxQuant. MS/MS spectra were searched against a database consisting of 10,152 protein entries from *S*. Typhimurium and 285 commonly observed contaminants. In a database search, full specificity was required for trypsin. Cleavage specificity C-terminal of phenylalanine, tryptophan, tyrosine, leucine, and methionine was set for chymotrypsin, and up to 5 missed cleavages were allowed. Carbamidomethylation of cysteine was set as fixed modification, protein N-terminal acetylation, and oxidation of methionine were set as variable modifications. Initial precursor mass tolerance was set to 4.5 parts per million (ppm) and at the fragment ion level 0.5 dalton (Da) was set for CID fragmentation. Peptide, protein, and modification site identifications were filtered using a target-decoy approach at a false discovery rate (FDR) set to 0.01 [59]. The mass spectrometry data have been deposited to the ProteomeXchange Consortium (http://proteomecentral.proteomexchange.org) via the PRIDE partner repository with the data set identifier PXD005597.

The numerical data used in all figures are included in S1 Data.

## Supporting information

S1 Fig Quality score assessment of de novo predictions of FliO, FliP, FliQ, and FliR homologs. Hits are sorted by decreasing score on the x-axis. Black dots represent previously annotated proteins, red dots the newly predicted ones. Darker shades of grey indicate increasing degree of homology.
(TIFF)

S2 Fig Fingerprint alignment of FliP homologs of flagellar T3SS and virulence-associated T3SS. Increasing shades of grey indicate the degree of amino acid conservation.
(TIFF)

S3 Fig Effects of excess FliP and FliO on motility and flagellin secretion. A. Motility of the wild type (WT) and a Δ*fliO* strain carrying pKG116-*fliO* (p*fliO*), pKG116-*fliP* (p*fliP*) or pKG116 empty vector control (VC) was analyzed in the presence or absence of inducer (1 μM and 10 μM NaSal). Expression of *fliO* from pKG116 is leaky also in the absence of inducer. Halo sizes were measured using ImageJ and expressed relative to the WT + VC in the absence of inducer. B. Flagellin secretion in the WT and the Δ*fliO* mutant harboring p*fliO*, p*fliP* or the empty vector control. Secreted flagellin was detected by Western blot using anti-FliC antibodies.
(TIFF)

S4 Fig Cross-complementation of a *fliO* mutant by overproduction of T3SS core integral membrane proteins. Δ*fliO* strains carrying pKG116-*fliO/P/Q/R*, pKG116-*flhB/A* or pKG116 empty vector control (VC) were incubated in LB + Cm for 5 h in the presence or absence of inducer (5 μM−10 μM NaSal). Expression of *fliO* from pKG116 is leaky also in the absence of inducer. Halo sizes were measured using ImageJ and expressed relative to the WT + VC without inducer.
(TIFF)

S5 Fig Motility phenotype of epitope tagged mutants. A. Example of motility in 0.3% agar at 37°C of strain harboring FliP-3×FLAG (WT, EM2225; Δ*fliO*, EM3201; Δ*fliF*, EM4909; Δ*fliF* Δ*fliO*, EM4910) or FliP-3×HA FliO-3×FLAG (WT, EM2269; Δ*fliF*, EM3910) chromosomal fusions. Relative motility and standard deviation 4 h and 7 h after inoculation are indicated below (n = 4). B. Shows representative example of motility of episomally encoding FliP-3×HA strains (WT, TH437; Δ*fliP*, TH17448; Δ*fliOP*, EM1610) grown 5 h at 37°C in LB + Cm + 0.3% agar non-induced (top row) or induced with 1 μM of NaSal (bottom row). Relative motility and standard deviation 5 h after inoculation are indicated below (n = 9).
(TIFF)

S6 Fig FliP degradation in FliO point mutants. Chromosomally encoded FliP_Q22-_3×HA protein levels were monitored at 0, 60, 120 and 180 min after protein synthesis arrest in the wild type (WT, TH17323) and FliO point mutants (FliO^L91R^, EM2742; FliO^V72G^, EM2743; FliO^ΔL91-L94^, EM2744). Western blot was performed using anti-HA antibody and DnaK was used as loading control.
(TIFF)

S7 Fig The absence of FliO does not affect FliP membrane integration. Membrane and cytosol fractions of the WT (EM2225) and Δ*fliO* mutant (EM3201) were collected 0 min and 120 min after synthesis arrest and separated by ultracentrifugation. Unwashed, solubilized membranes, urea-washed membranes and precipitated cytosols were separated on a 15% SDS-PAGE and FliP protein was detected by Western blot using anti-FLAG antibodies. TCE staining shows total protein levels. DnaK was used as cytosolic protein control.
(TIFF)

S8 Fig Stability of FliP upon overproduction of YccA, an inhibitor of FtsH. A. Bacterial growth analysis to test the functionality of the pTrc99a-YccA(STM1085)_Δ__aa5-12_ expression plasmid. Expression of YccA from a moderate copy plasmid by addition of 1 mM IPTG rescued the lethal growth defect of a LamB_181_-LacZ hybrid (EM6396, pEM3191 (YccA); EM6399, pTrc99AFF4 vector control (VC)) in the presence of maltose due to jamming of the Sec-translocon. A short LamB_26_-LacZ fusion (EM6394, pEM3191 (YccA); EM6397, pTrc99AFF4 vector control (VC)) is not targeted to the Sec translocon and bacterial growth is not affected by addition of maltose (Mal). B. The wild type (WT, TH17323) and Δ*fliO* mutant harboring a IPTG-inducible pTrc99a empty vector control (VC, EM3192) and pTrc99a-YccA_Δ__aa5-12_ (YccA, EM3193) were grown in LB + Amp. Expression of YccA was induced by addition of 1 mM IPTG. Samples were taken at 0 and 180 min after synthesis stop and separated on 15% SDS-PAGE. FliP_Q22_-3×HA protein was detected by Western blot using anti-HA antibodies. TCE staining indicates total protein levels.
(TIFF)

S9 Fig Flagellin secretion in HaloTag fusion strains. FliC locked strains (FliN-HaloTag, EM1330; FliN-SNAP-tag, EM1331; FliM-HaloTag, EM1328; FliM-SNAP-tag, EM1329; FliO-HaloTag, EM1326; FliO-SNAP-tag, EM1327; Δ*fliO*, EM2272, wild type (WT), TH5861) were grown in LB at 37°C. Cells (top) and supernatants (bottom) were harvested and separated by centrifugation. Samples were analyzed by Western blot using anti-FliC antibodies. TCE staining was used to determine total protein levels.
(TIFF)

S10 Fig Analysis of co-localization of FliO and flagellar basal body complexes by structured illumination microscopy (SIM). The sub-cellular co-localization of chromosomal FliO-HaloTag (EM1204, EM1214) and FliN-HaloTag (EM3202) with hook-basal-body components was analyzed using structured illumination microscopy. Strain EM1204 additionally harbored a chromosomal FliM-mEos fusion. Strains EM1214 and EM3202 additionally harbored a chromosomal epitope-tagged variant of the hook protein (FlgE-3×HA). Scale bar 2 μm.
(TIFF)

S11 Fig LC-MS/MS analysis of FliP containing complexes separated by BN-PAGE. A. LC-MS/MS analysis of prominent FliP-containing complexes revealed after BN-PAGE separation. The heat map represents the relative abundance of peptides from relevant proteins detected in the indicated FliP-containing complexes. B. LC-MS/MS analysis of prominent FliP-containing bands after immunoprecipitation of chromosomal FliP-3xFLAG in a rod^−^ (Δ*flgBC*) strain background. The heat map represents the relative abundance of peptides from relevant proteins detected in the indicated FliP-containing complexes.
(TIFF)

S12 Fig BN-PAGE analysis of FlhA-containing complexes. Anti-FlhA (left panel) and anti-HA (middle panel) Western blot of BN-PAGE of crude membrane extracts prepared from the WT (TH17323), Δ*fliO* (EM1274), Δ*fliF* (EM3910), and Δ*fliO* Δ*fliF* (EM1618) mutant strains encoding for chromosomal FliP-3×HA. The merged anti-FlhA and anti-HA Western blots are shown in the right panel.
(TIFF)

S13 Fig Co-purification of FliO and FliP. A. Immunoprecipitation of chromosomal FliP-3xFLAG. LC-MS/MS analysis on the indicated FliP-containing bands was performed and the results are summarized in S11B Fig. B. Immunoprecipitation of chromosomal FliO-3xFLAG specifically pulls down FliP. C. Co-purification of FliO and FliP complexes. FliO-FliP_His6_ was expressed in *Escherichia coli* and affinity purified after solubilisation of crude membrane extracts in DDM. Top: size exclusion chromatography after FliP_His6_ affinity purification and electron microscopy analysis of indicated elution fractions. Bottom: SDS-PAGE analysis of input and size exclusion chromatography elution fractions. Proteins were visualized using coomassie staining and polyclonal anti-FliP / anti-FliO Western blot analysis.
(TIFF)

S1 Table List of strains and plasmids used in this study. All strains are derivative of Salmonella enterica servovar Typhimurium LT2 unless noted otherwise.
(DOCX)

S1 Data Numerical data used in all figures. (XLSX)

## Abbreviations

BN-PAGE: blue native PAGE
C ring: cytoplasmic ring
DDM: n-dodecyl-β-D-maltoside
dSTORM: direct stochastic optical reconstruction microscopy
fT3SS: flagellar-specific type III secretion system
HA: 3×hemagglutinin
HBB: hook-basal-body
HMM: Hidden Markov Model
LB: lysogeny broth
LC-MS/MS: liquid chromatography-tandem mass spectrometry
MSD: Mean square displacement
Pfam: protein family
pmf: proton motive force
SIM: structured illumination microscopy
SMT: Single-molecule tracking
T3SS: type III secretion system
TIRF: total internal reflection fluorescence
VC: vector control
vT3SS: virulence-associated type III secretion system
WT: wild type
