# Supplementary figures and images for: A flagellum-specific chaperone facilitates assembly of the core type III export apparatus of the bacterial flagellum
Source: PLoS Biol. 2017 Aug 3;15(8):e2002267. doi: 10.1371/journal.pbio.2002267 (PMC5542435; doi:10.1371/journal.pbio.2002267)

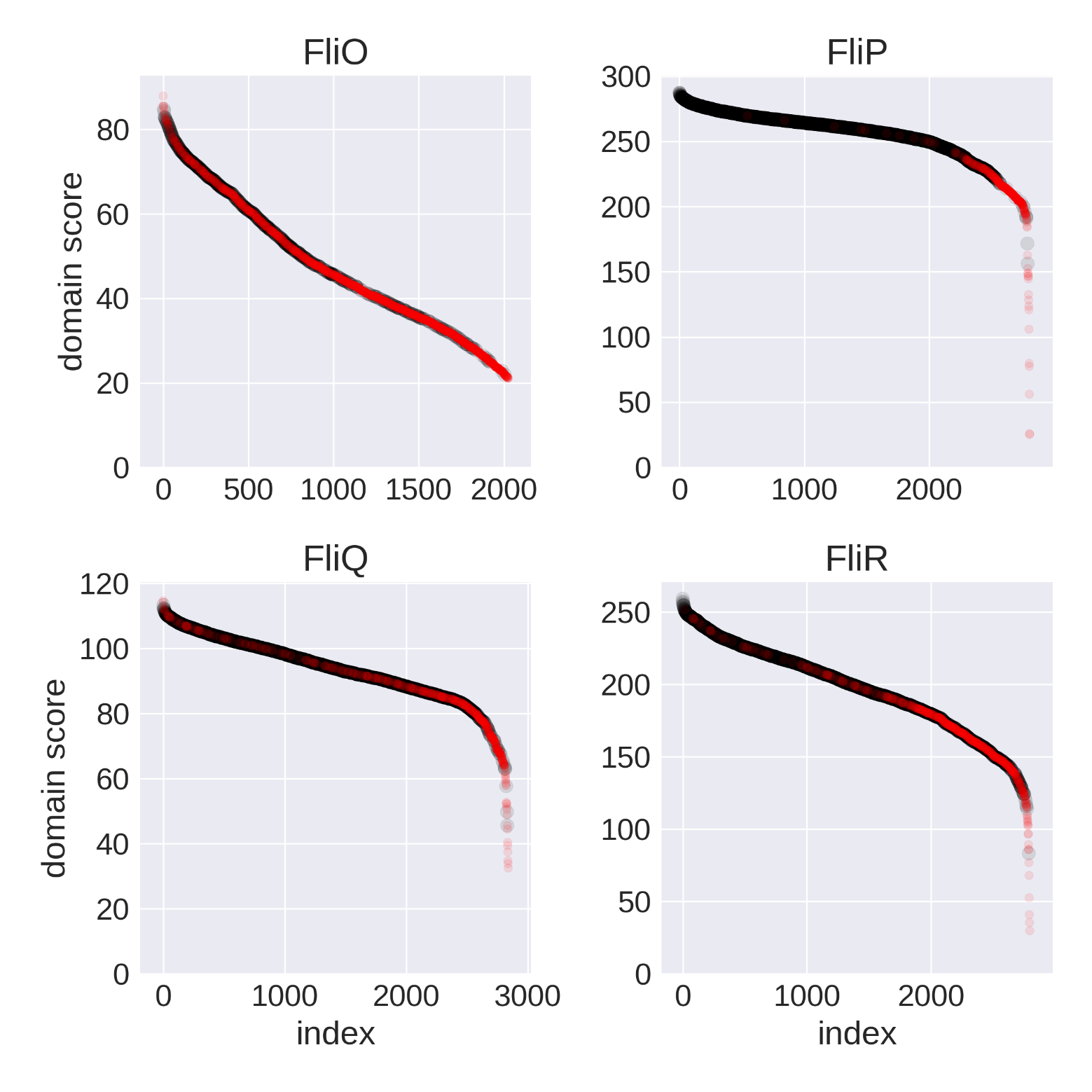

Supplement: S1 Fig — Hits are sorted by decreasing score on the x-axis. Black dots represent previously annotated proteins, red dots the newly predicted ones. Darker shades of grey indicate increasing degree of homology. (TIFF) [file pbio.2002267.s001.tiff]

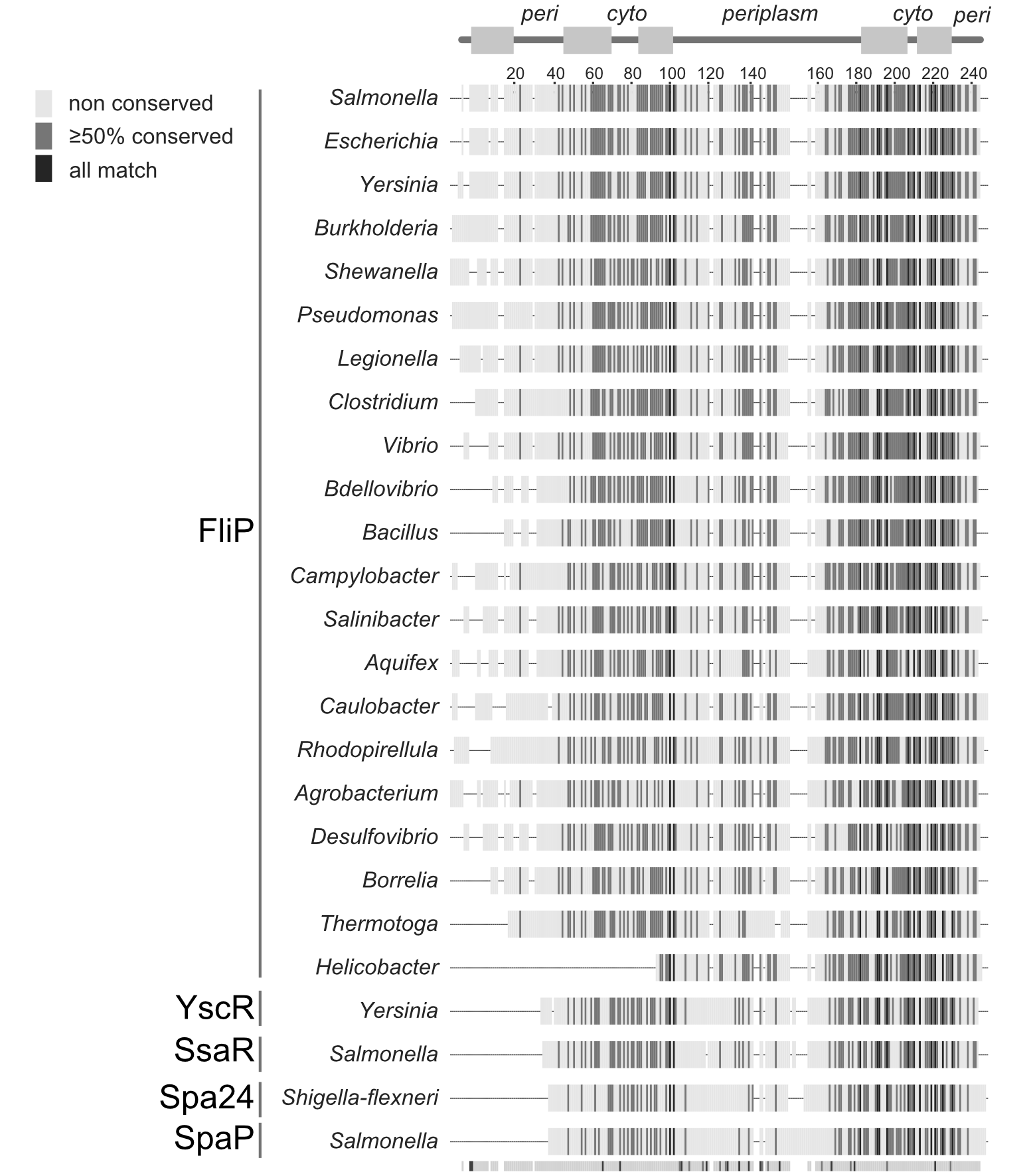

Supplement: S2 Fig — Increasing shades of grey indicate the degree of amino acid conservation. (TIFF) [file pbio.2002267.s002.tiff]

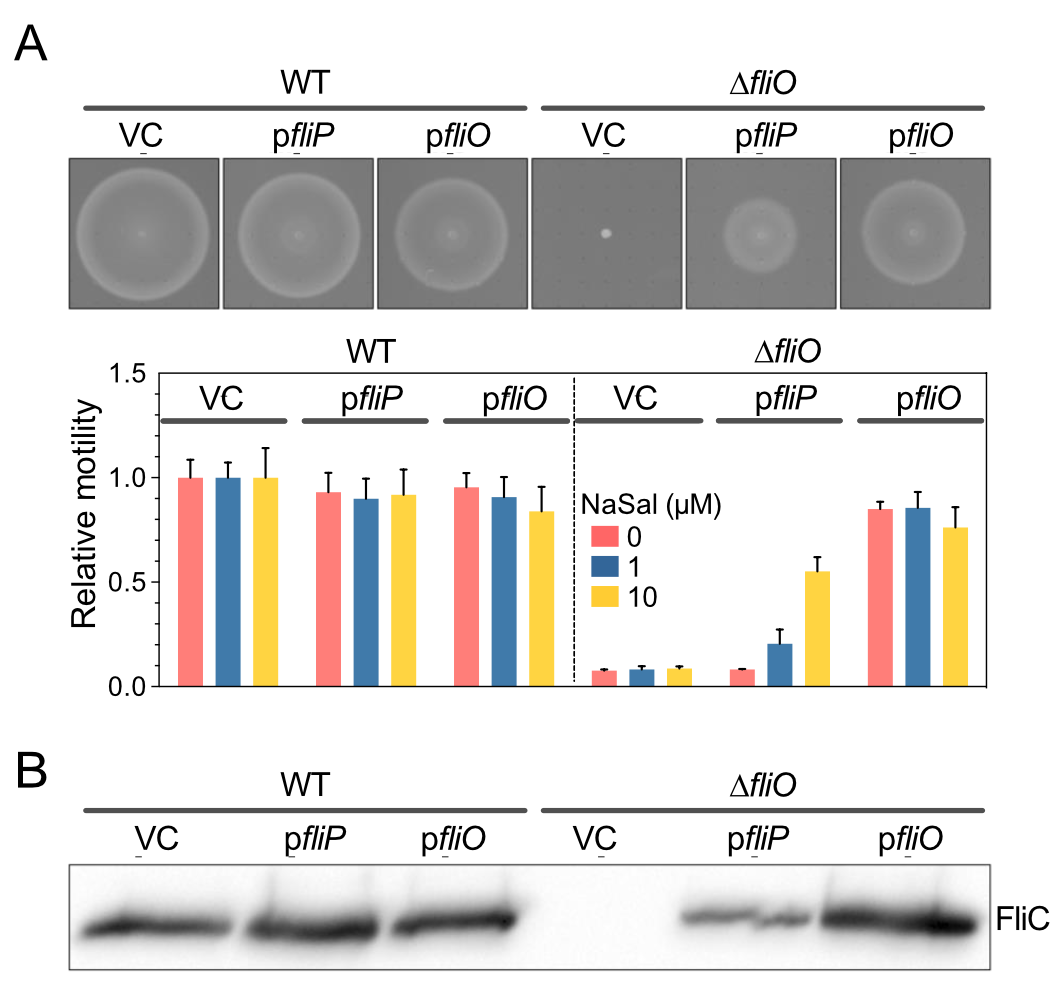

Supplement: S3 Fig — A. Motility of the wild type (WT) and a ΔfliO strain carrying pKG116-fliO (pfliO), pKG116-fliP (pfliP) or pKG116 empty vector control (VC) was analyzed in the presence or absence of inducer (1 μM and 10 μM NaSal). Expression of fliO from pKG116 is leaky also in the absence of inducer. Halo sizes were measured using ImageJ and expressed relative to the WT + VC in the absence of inducer. B. Flagellin secretion in the WT and the ΔfliO mutant harboring pfliO, pfliP or the empty vector control. Secreted flagellin was detected by Western blot using anti-FliC antibodies. (TIFF) [file pbio.2002267.s003.tiff]

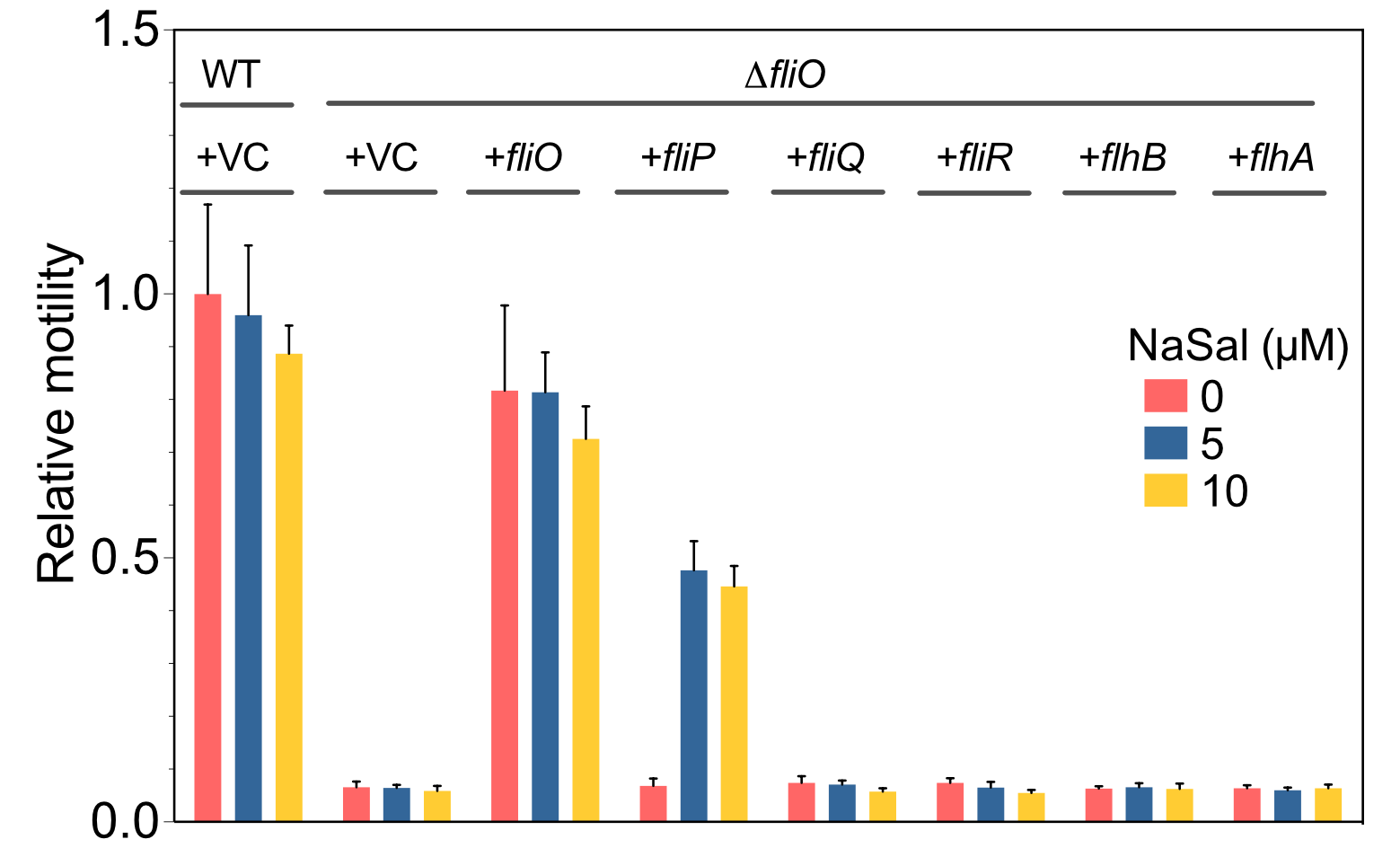

Supplement: S4 Fig — ΔfliO strains carrying pKG116-fliO/P/Q/R, pKG116-flhB/A or pKG116 empty vector control (VC) were incubated in LB + Cm for 5 h in the presence or absence of inducer (5 μM−10 μM NaSal). Expression of fliO from pKG116 is leaky also in the absence of inducer. Halo sizes were measured using ImageJ and expressed relative to the WT + VC without inducer. (TIFF) [file pbio.2002267.s004.tiff]

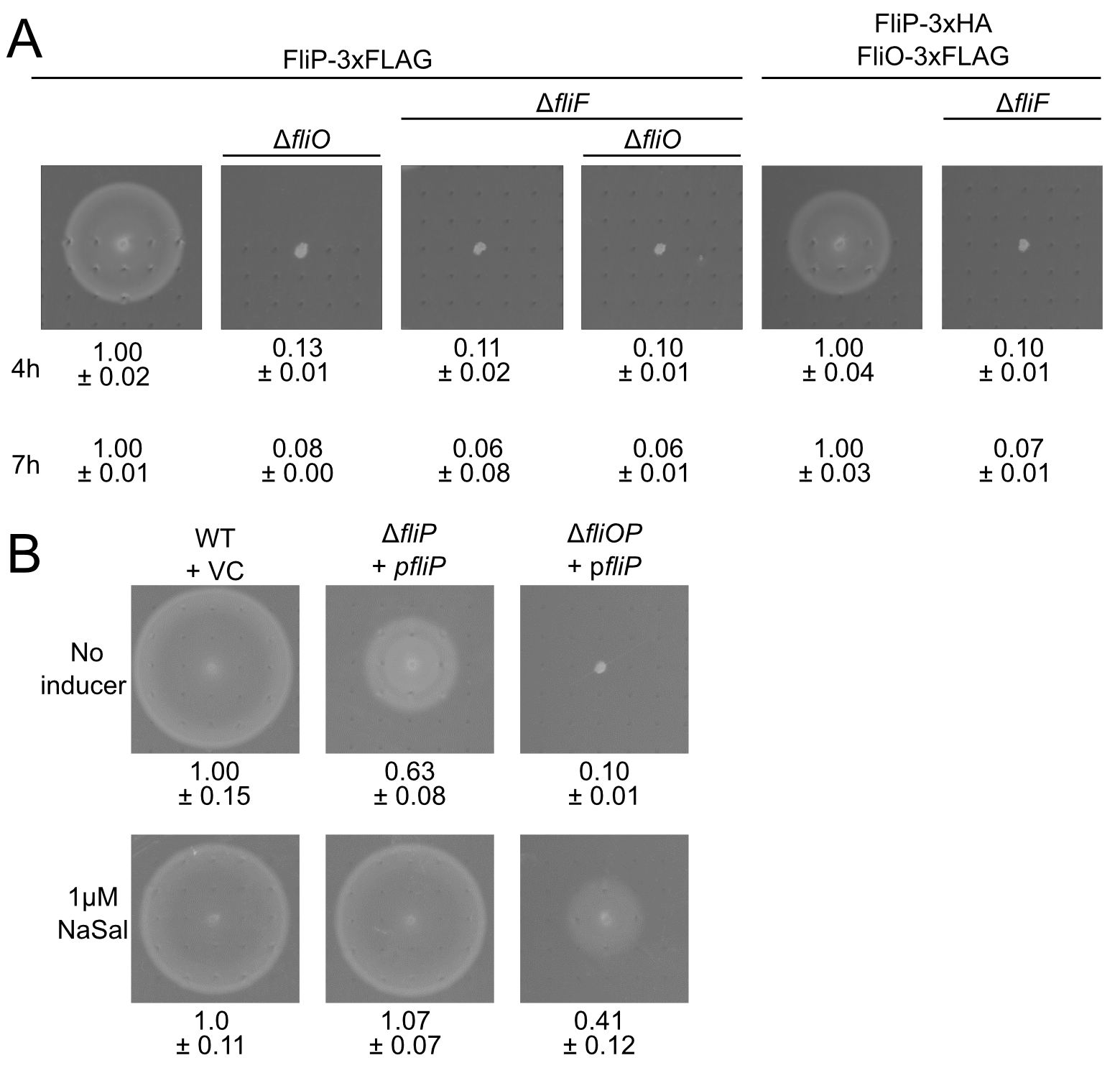

Supplement: S5 Fig — A. Example of motility in 0.3% agar at 37°C of strain harboring FliP-3×FLAG (WT, EM2225; ΔfliO, EM3201; ΔfliF, EM4909; ΔfliF ΔfliO, EM4910) or FliP-3×HA FliO-3×FLAG (WT, EM2269; ΔfliF, EM3910) chromosomal fusions. Relative motility and standard deviation 4 h and 7 h after inoculation are indicated below (n = 4). B. Shows representative example of motility of episomally encoding FliP-3×HA strains (WT, TH437; ΔfliP, TH17448; ΔfliOP, EM1610) grown 5 h at 37°C in LB + Cm + 0.3% agar non-induced (top row) or induced with 1 μM of NaSal (bottom row). Relative motility and standard deviation 5 h after inoculation are indicated below (n = 9). (TIFF) [file pbio.2002267.s005.tiff]

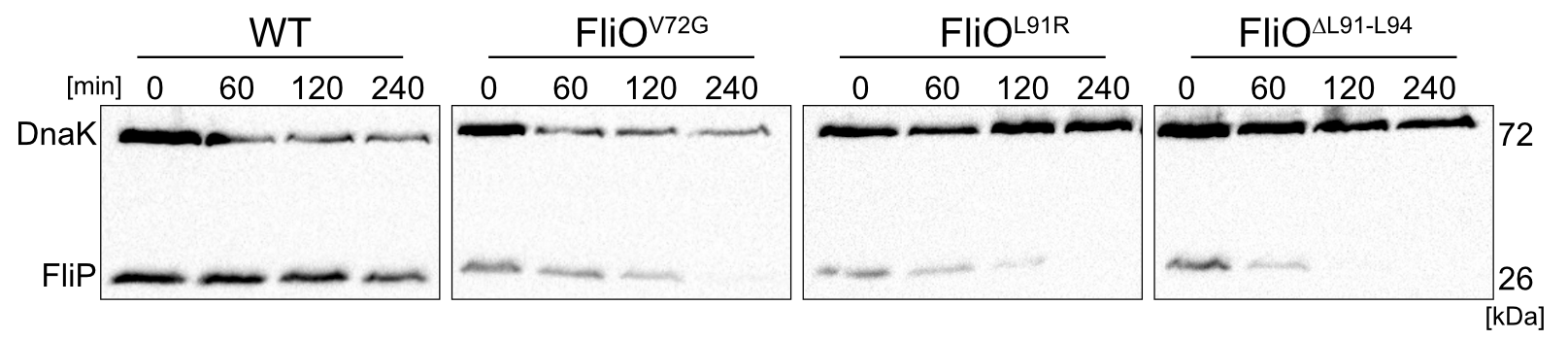

Supplement: S6 Fig — Chromosomally encoded FliPQ22-3×HA protein levels were monitored at 0, 60, 120 and 180 min after protein synthesis arrest in the wild type (WT, TH17323) and FliO point mutants (FliOL91R, EM2742; FliOV72G, EM2743; FliOΔL91-L94, EM2744). Western blot was performed using anti-HA antibody and DnaK was used as loading control. (TIFF) [file pbio.2002267.s006.tiff]

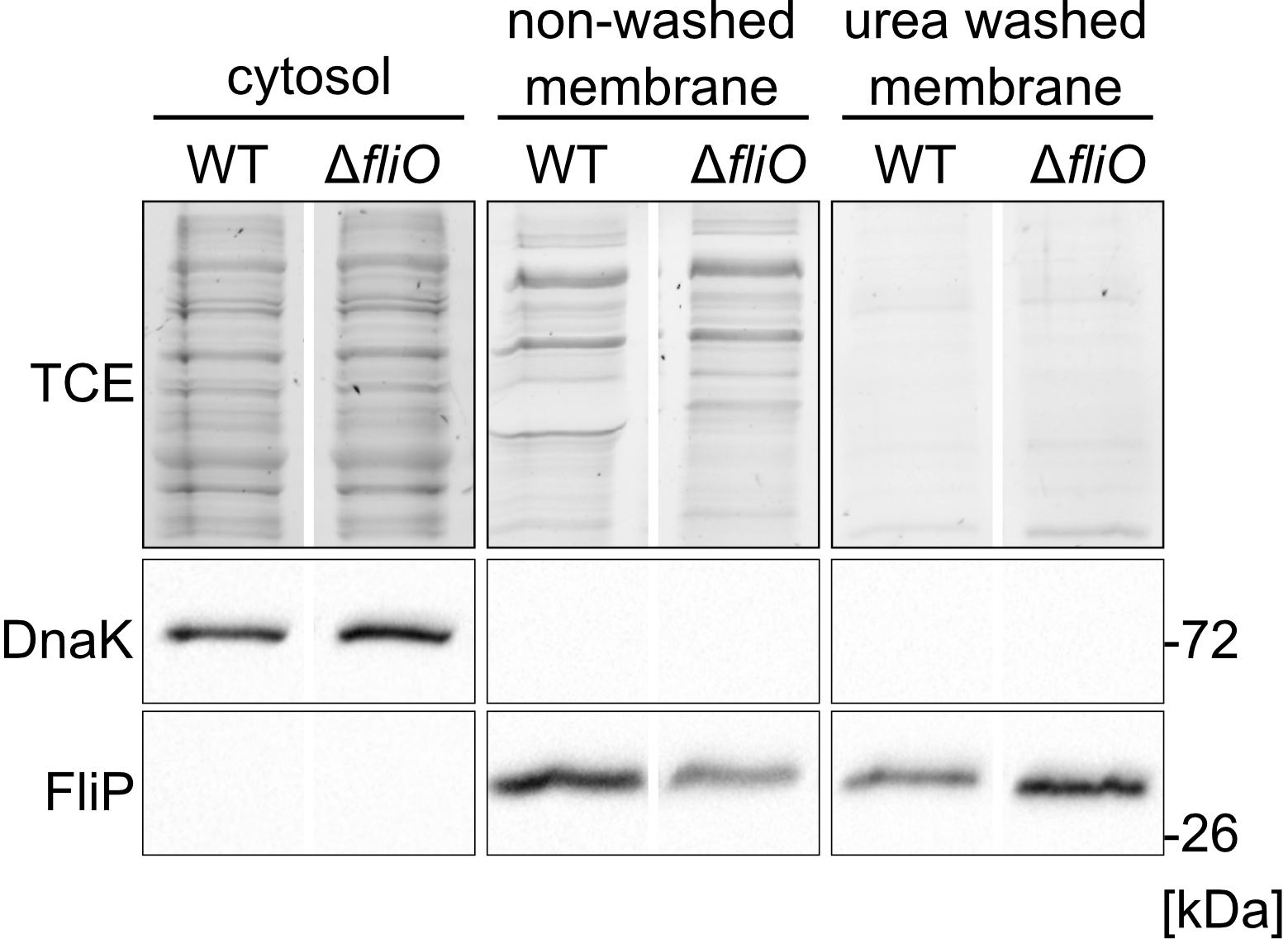

Supplement: S7 Fig — Membrane and cytosol fractions of the WT (EM2225) and ΔfliO mutant (EM3201) were collected 0 min and 120 min after synthesis arrest and separated by ultracentrifugation. Unwashed, solubilized membranes, urea-washed membranes and precipitated cytosols were separated on a 15% SDS-PAGE and FliP protein was detected by Western blot using anti-FLAG antibodies. TCE staining shows total protein levels. DnaK was used as cytosolic protein control. (TIFF) [file pbio.2002267.s007.tiff]

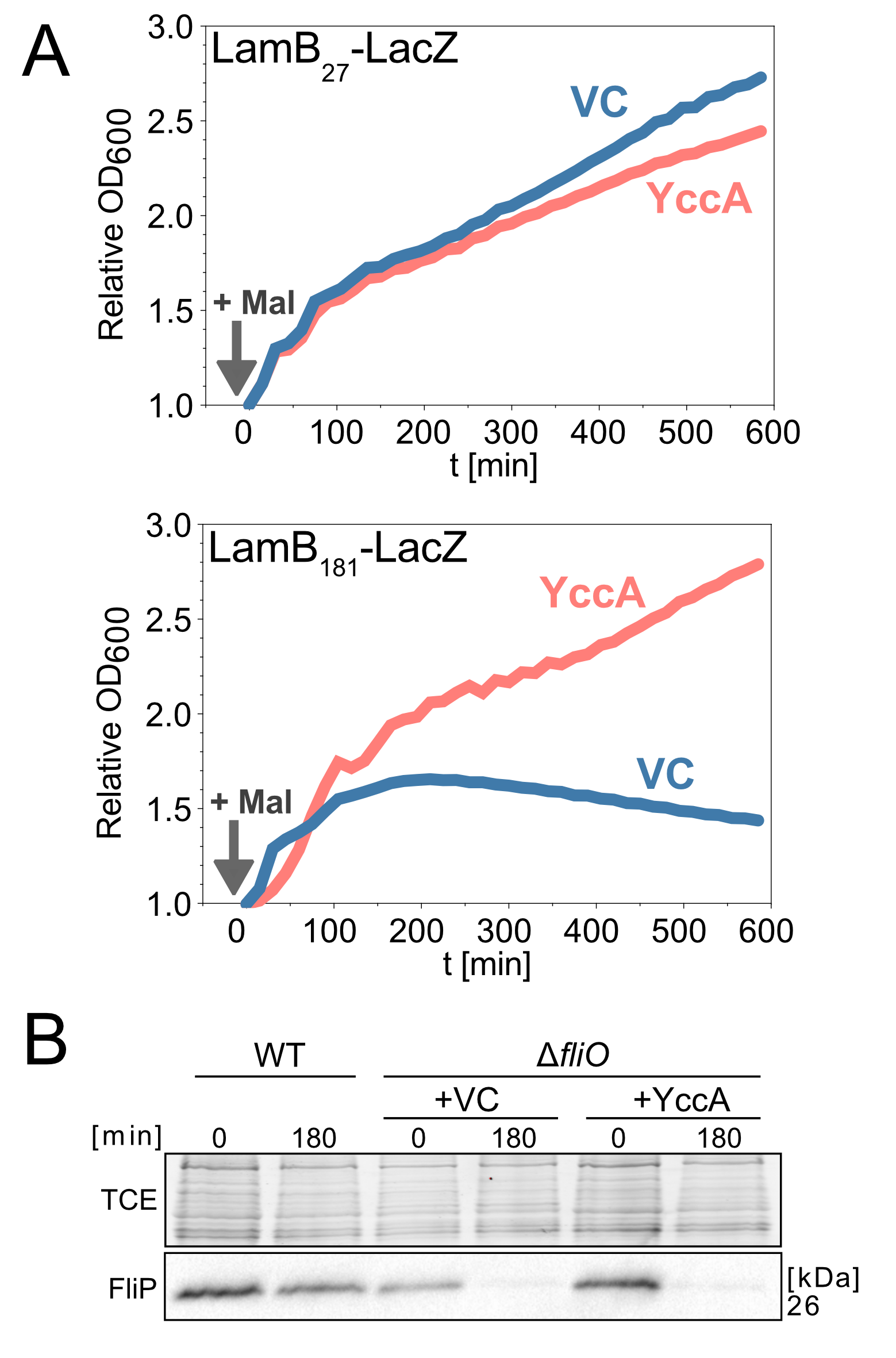

Supplement: S8 Fig — A. Bacterial growth analysis to test the functionality of the pTrc99a-YccA(STM1085)Δaa5-12 expression plasmid. Expression of YccA from a moderate copy plasmid by addition of 1 mM IPTG rescued the lethal growth defect of a LamB181-LacZ hybrid (EM6396, pEM3191 (YccA); EM6399, pTrc99AFF4 vector control (VC)) in the presence of maltose due to jamming of the Sec-translocon. A short LamB26-LacZ fusion (EM6394, pEM3191 (YccA); EM6397, pTrc99AFF4 vector control (VC)) is not targeted to the Sec translocon and bacterial growth is not affected by addition of maltose (Mal). B. The wild type (WT, TH17323) and ΔfliO mutant harboring a IPTG-inducible pTrc99a empty vector control (VC, EM3192) and pTrc99a-YccAΔaa5-12 (YccA, EM3193) were grown in LB + Amp. Expression of YccA was induced by addition of 1 mM IPTG. Samples were taken at 0 and 180 min after synthesis stop and separated on 15% SDS-PAGE. FliPQ22-3×HA protein was detected by Western blot using anti-HA antibodies. TCE staining indicates total protein levels. (TIFF) [file pbio.2002267.s008.tiff]

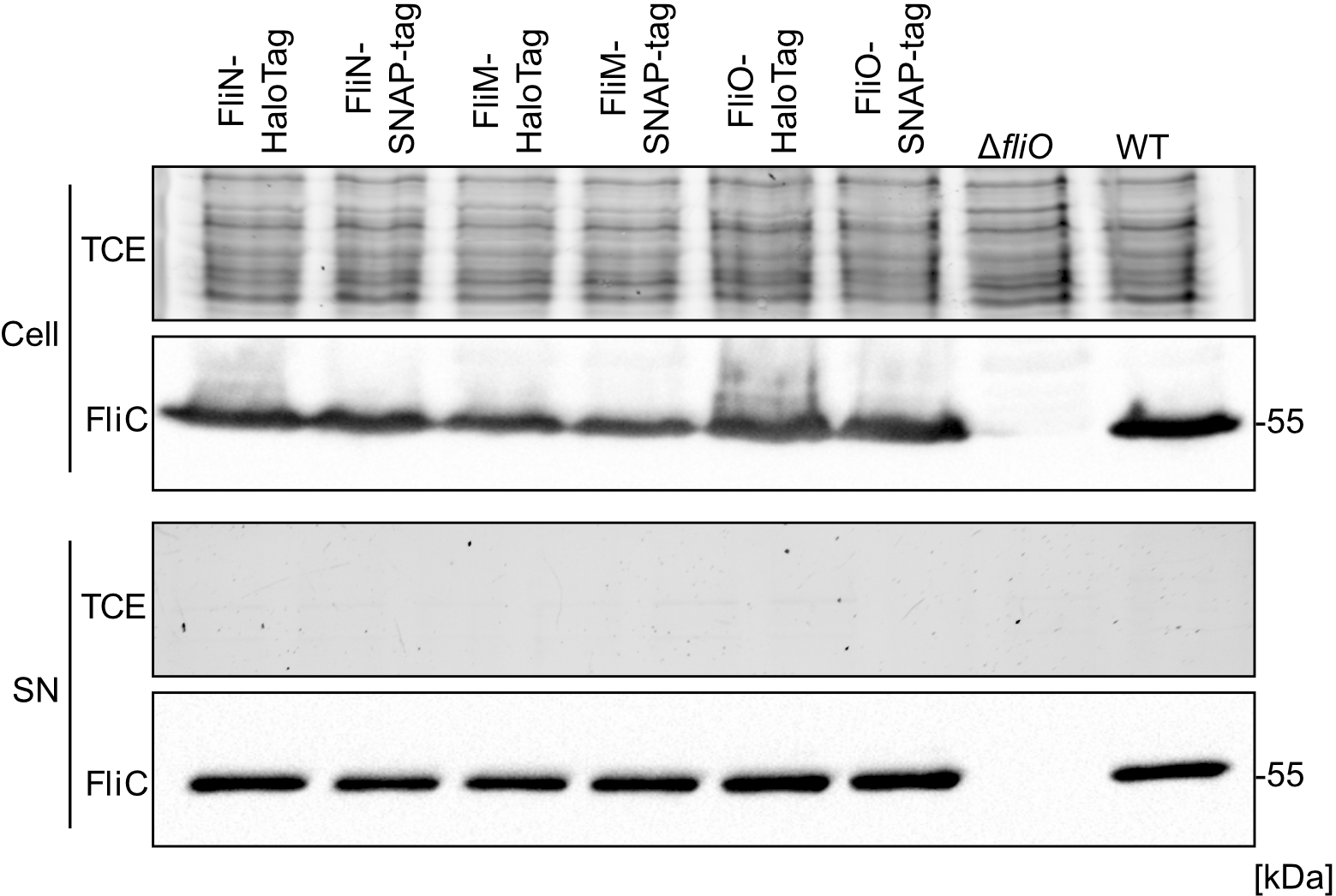

Supplement: S9 Fig — FliC locked strains (FliN-HaloTag, EM1330; FliN-SNAP-tag, EM1331; FliM-HaloTag, EM1328; FliM-SNAP-tag, EM1329; FliO-HaloTag, EM1326; FliO-SNAP-tag, EM1327; ΔfliO, EM2272, wild type (WT), TH5861) were grown in LB at 37°C. Cells (top) and supernatants (bottom) were harvested and separated by centrifugation. Samples were analyzed by Western blot using anti-FliC antibodies. TCE staining was used to determine total protein levels. (TIFF) [file pbio.2002267.s009.tiff]

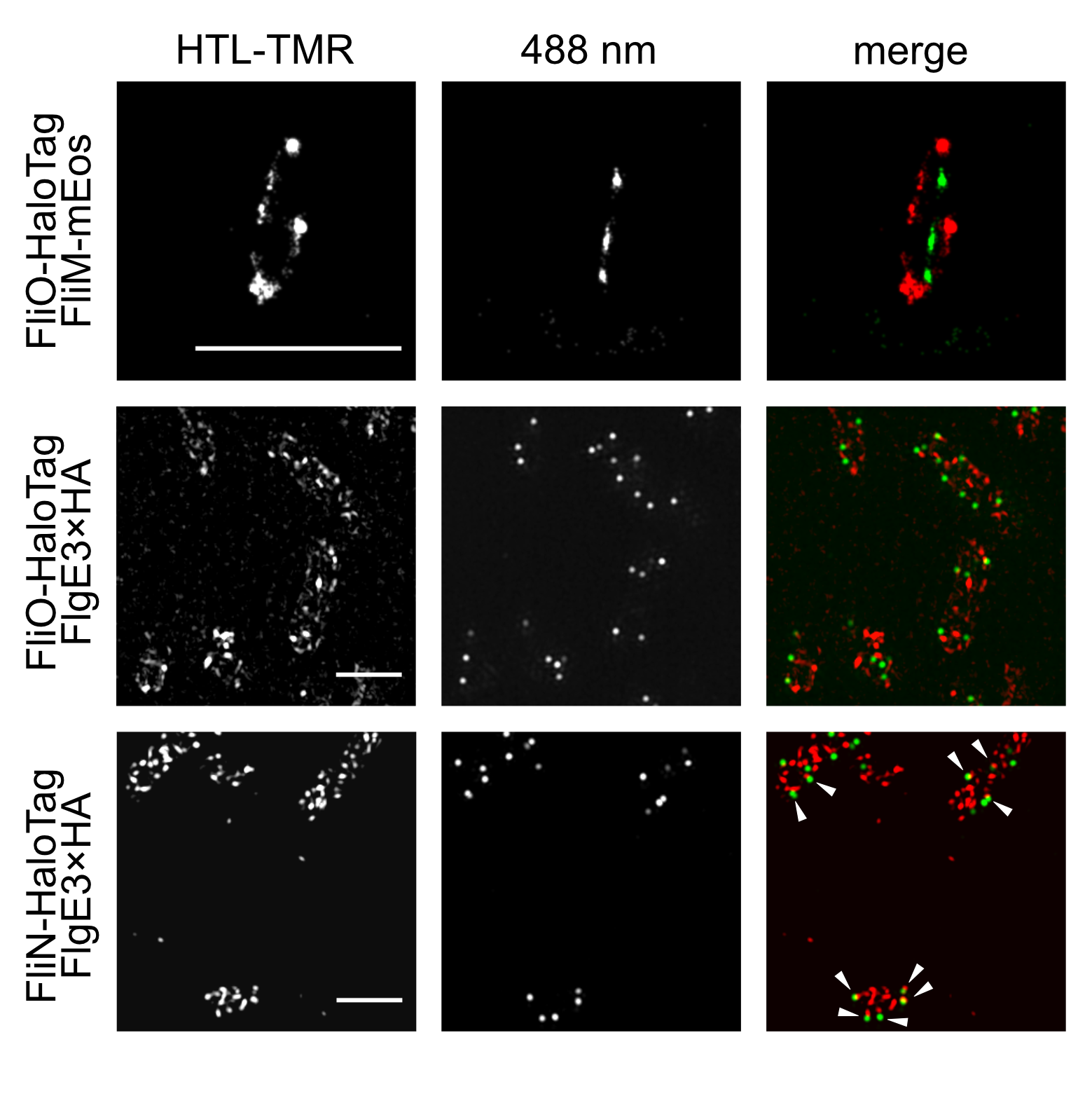

Supplement: S10 Fig — The sub-cellular co-localization of chromosomal FliO-HaloTag (EM1204, EM1214) and FliN-HaloTag (EM3202) with hook-basal-body components was analyzed using structured illumination microscopy. Strain EM1204 additionally harbored a chromosomal FliM-mEos fusion. Strains EM1214 and EM3202 additionally harbored a chromosomal epitope-tagged variant of the hook protein (FlgE-3×HA). Scale bar 2 μm. (TIFF) [file pbio.2002267.s010.tiff]

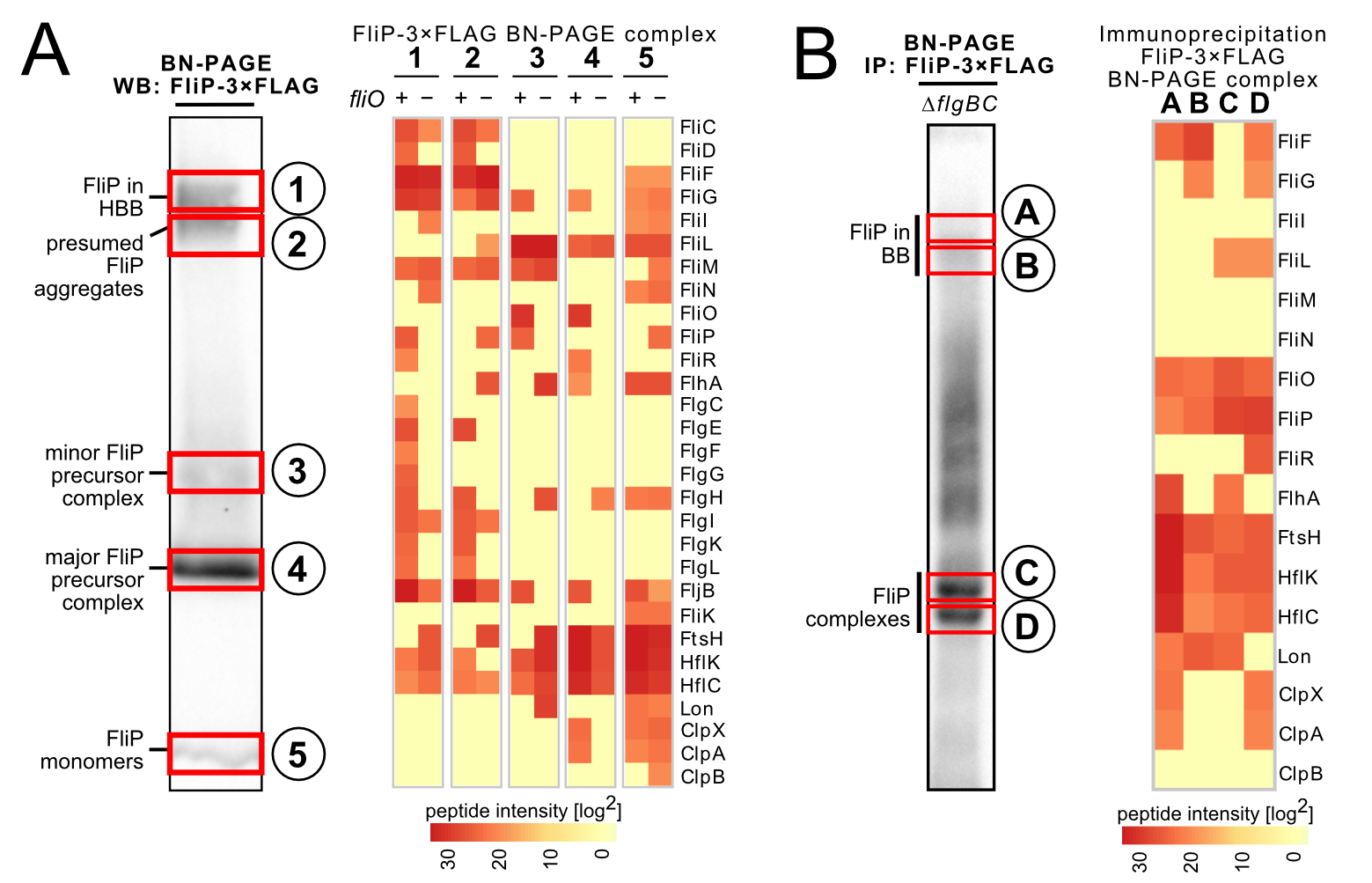

Supplement: S11 Fig — A. LC-MS/MS analysis of prominent FliP-containing complexes revealed after BN-PAGE separation. The heat map represents the relative abundance of peptides from relevant proteins detected in the indicated FliP-containing complexes. B. LC-MS/MS analysis of prominent FliP-containing bands after immunoprecipitation of chromosomal FliP-3xFLAG in a rod− (ΔflgBC) strain background. The heat map represents the relative abundance of peptides from relevant proteins detected in the indicated FliP-containing complexes. (TIFF) [file pbio.2002267.s011.tiff]

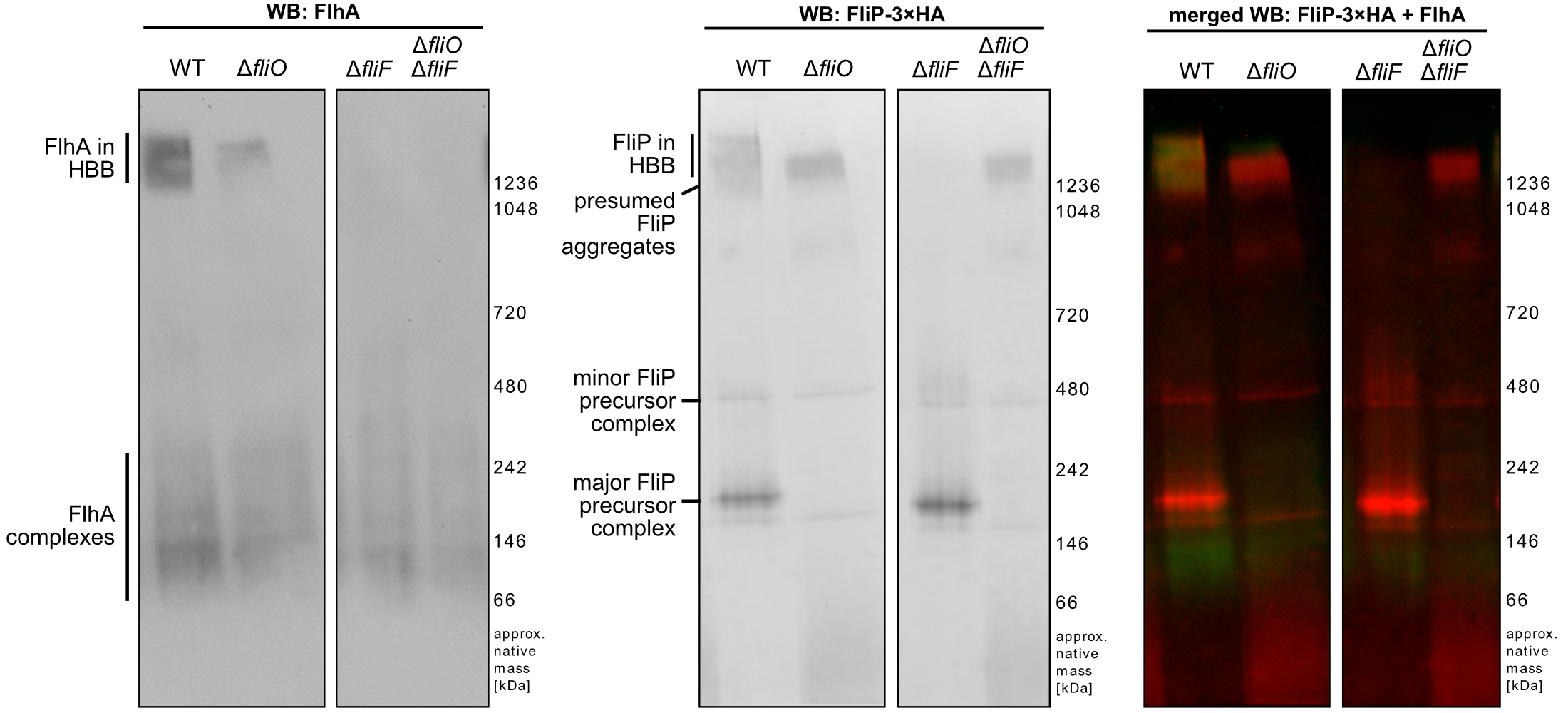

Supplement: S12 Fig — Anti-FlhA (left panel) and anti-HA (middle panel) Western blot of BN-PAGE of crude membrane extracts prepared from the WT (TH17323), ΔfliO (EM1274), ΔfliF (EM3910), and ΔfliO ΔfliF (EM1618) mutant strains encoding for chromosomal FliP-3×HA. The merged anti-FlhA and anti-HA Western blots are shown in the right panel. (TIFF) [file pbio.2002267.s012.tiff]

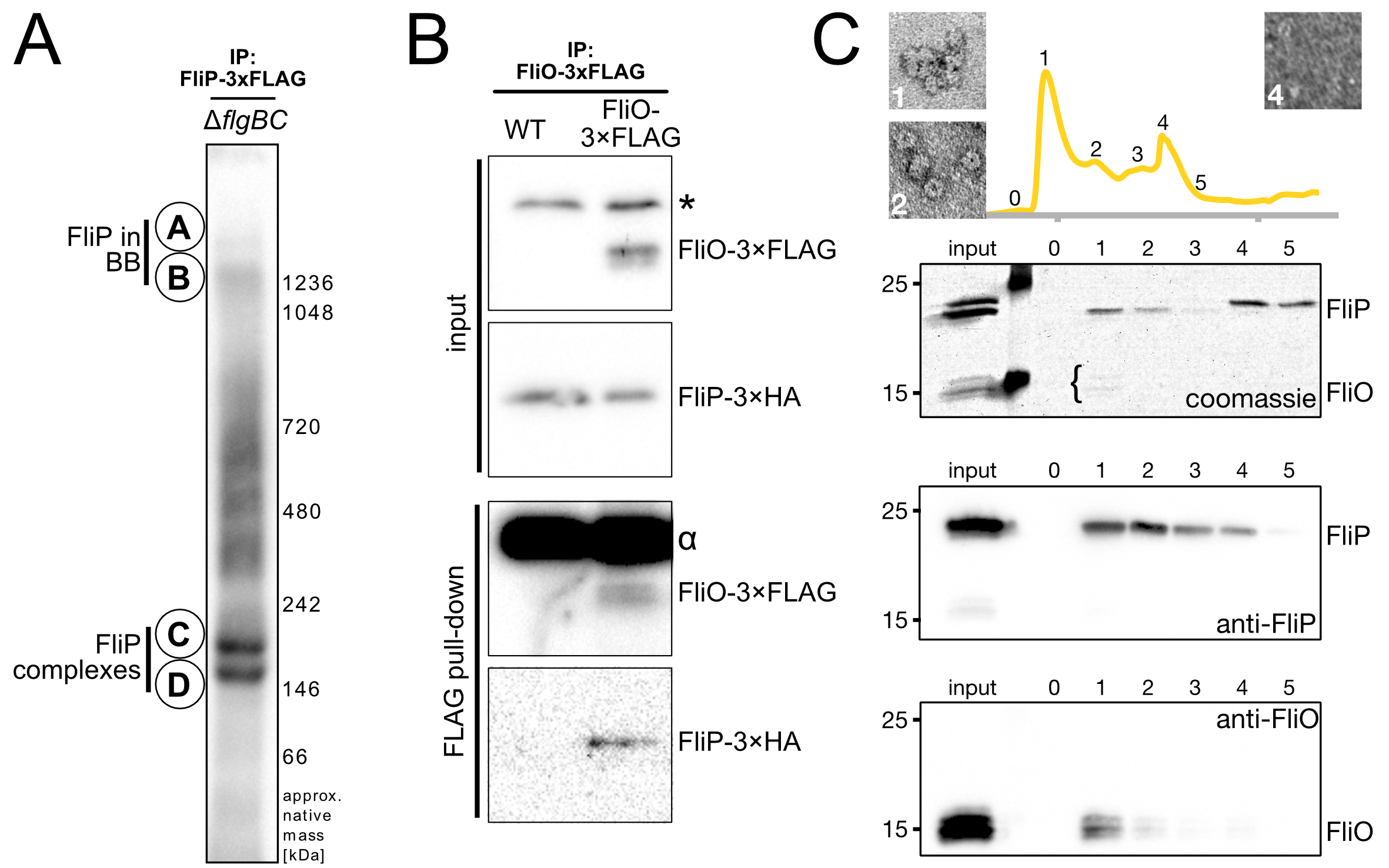

Supplement: S13 Fig — A. Immunoprecipitation of chromosomal FliP-3xFLAG. LC-MS/MS analysis on the indicated FliP-containing bands was performed and the results are summarized in S11B Fig. B. Immunoprecipitation of chromosomal FliO-3xFLAG specifically pulls down FliP. C. Co-purification of FliO and FliP complexes. FliO-FliPHis6 was expressed in Escherichia coli and affinity purified after solubilisation of crude membrane extracts in DDM. Top: size exclusion chromatography after FliPHis6 affinity purification and electron microscopy analysis of indicated elution fractions. Bottom: SDS-PAGE analysis of input and size exclusion chromatography elution fractions. Proteins were visualized using coomassie staining and polyclonal anti-FliP / anti-FliO Western blot analysis. (TIFF) [file pbio.2002267.s013.tiff]
