# Supplementary material for: A flagellum-specific chaperone facilitates assembly of the core type III export apparatus of the bacterial flagellum
Source: PLoS Biol. 2017 Aug 3;15(8):e2002267. doi: 10.1371/journal.pbio.2002267 (PMC5542435; doi:10.1371/journal.pbio.2002267)
Supplement: S1 Table — All strains are derivative of Salmonella enterica servovar Typhimurium LT2 unless noted otherwise. (DOCX) [file pbio.2002267.s014.docx]

| Strain | **Relevant genotype** | **Reference** |
| --- | --- | --- |
| BL21(DE3) | *Escherichia coli* host for overexpression from the T7 promotor | Novagen |
| TH437 | LT2 wild type | K.T. Hughes |
| TH5861 | Δ*hin*-5717::Frt-Chloramphenical acetyl transferase-Frt (FCF) | K.T. Hughes |
| TH10605 | *fliO*1087_L91R_ *fljB*enx vh2 | S. Yamaguchi |
| TH10607 | *fliO*1089_V72G_ *fljB*enx vh2 | S. Yamaguchi |
| TH10608 | *fliO*1432_∆L91-L94_ *fljB*enx vh2 | S. Yamaguchi |
| TH10548 | *∆fliO6708* | this study |
| TH17323 | *fliP*8004::Q_22_-3×HA | this study |
| TH17448 | Δ*fliP*6709 / pKG116-*fliP*::Q_22_-3×HA | this study |
| EM1077 | *fliO*22315::HaloTag | this study |
| EM1081 | *fliN*22319::HaloTag | ([1](#_ENREF_1)) |
| EM1204 | *fliM*7941::mEOS *fliO*22315::HaloTag | this study |
| EM1214 | *fliO*22315::HaloTag *flgE*7742::3×HA | this study |
| EM1274 | Δ*fliO*22334 *fliP*8004::Q_22_-3×HA | this study |
| EM1326 | *fliO*22315::HaloTag Δ*hin*-5717::FCF | this study |
| EM1327 | *fliO*22316::SNAP-tag Δ*hin*-5717::FCF | this study |
| EM1328 | *fliM*22317::HaloTag Δ*hin*-5717::FCF | this study |
| EM1329 | *fliM*22318::SNAP-tag Δ*hin*-5717::FCF | this study |
| EM1330 | *fliN*22319::HaloTag Δ*hin*-5717::FCF | ([1](#_ENREF_1)) |
| EM1331 | *fliN*22320::SNAP-tag Δ*hin*-5717::FCF | this study |
| EM1609 | Δ*fliOP*6856 / pKG116 | this study |
| EM1610 | Δ*fliOP*6856 / pKG116-*fliP*::Q_22-_3×HA | this study |
| EM1618 | *fliF*22328*::tetRA ∆fliO*22334 *fliP*8004::Q_22_-3×HA | this study |
| EM2225 | *fliP*22354::G_157_-×FLAG | this study |
| EM2272 | *∆fliO6708* Δ*hin*-5717::FCF | this study |
| EM2269 | *fliO*22348::3×FLAG *fliP*8004::Q_22_-3×HA | this study |
| EM2320 | Δ*hin*-5717::FRT / pKG116 | this study |
| EM2321 | Δ*hin*-5717::FRT / pKG116-*fliP* | this study |
| EM2322 | Δ*hin*-5717::FRT / pKG116-*fliO* | this study |
| EM2323 | Δ*fliO*6708 Δ*hin*-5717::FRT / pKG116 | this study |
| EM2324 | Δ*fliO*6708 Δ*hin*-5717::FRT / pKG116-*fliP* | this study |
| EM2325 | Δ*fliO*6708 Δ*hin*-5717::FRT / pKG116-*fliO* | this study |
| EM2640 | *fliN*22319::HaloTag ∆*fliF*5629::Frt-aminoglycoside phosphotransferase (Kanamycin resistance)-Frt (FKF) | this study |
| EM2716 | *fliP*22354::G_157_-3×FLAG *flgE*6569::*bla* Δ*flgBC*6557 Δ*ydiV* | this study |
| EM2742 | *fliO*1087_L91R_ *fliP*8004::Q_22_-3×HA | this study |
| EM2743 | *fliO*1089_V72G_ *fliP*8004::Q_22_-3×HA | this study |
| EM2744 | *fliO*1432_∆L91-L94_ *fliP*8004::Q_22_-3×HA | this study |
| EM3201 | Δ*fliO*22770 *fliP*22354::G_157_-3×Flag | this study |
| EM3202 | *fliN*22319::HaloTag *flgE*7742::3×HA | this study |
| Strain | **Relevant genotype** | **Reference** |
| EM3192 | Δ*fliO*22334 *fliP*8004::Q_22_-3×HA / pTrc99AFFA | this study |
| EM3193 | Δ*fliO*22334 *fliP*8004::Q_22_-3×HA / pTrc99FFA-STM1085_∆aa5-12_ | this study |
| EM3910 | Δ*fliF*7355 *fliO*22348::3×FLAG *fliP*8004::Q_22_-3×HA | this study |
| EM4018 | *fliP*8004::Q_22_-3×HA Δ*clpXP*::FRT | this study |
| EM4019 | Δ*fliO*22334 *fliP*8004::Q_22_-3×HA Δ*clpXP*::FRT | this study |
| EM4478 | *fliO*22348::3×FLAG *fliP*8004::Q_22_-3×HA Δ*lon* | this study |
| EM4479 | Δ*fliO*22334 *fliP*8004::Q_22_-3×HA Δ*lon* | this study |
| EM4859 | ∆*fliF7339::tetRA* *fliP*22354::G_157_-3×FLAG | this study |
| EM4909 | Δ*fliF*5629::FKF *fliP*22354::G_157_-3×FLAG | this study |
| EM4910 | Δ*fliF*5629::FKF Δ*fliO*22770 *fliP*22354::G_157_-3×FLAG | this study |
| EM6195 | ∆*fliF*7339::*tetRA* *fliO*::3×FLAG *fliP*22354::G_157_-3×FLAG *fliR*22357::TwinStrep ∆*ydiV*::FKF | this study |
| EM6196 | ∆*fliF*7339::*tetRA* *fliO*::3×FLAG *fliP*::Q_22_-3×HA *fliR22356*::3×FLAG ∆*ydiV*::FKF | this study |
| EM6221 | *fliP*22354::G_157_-3×FLAG ∆*flgBC*6143::*tetRA* | this study |
| EM6222 | ∆*fliO*22770 *fliP*22354::G_157_-3×FLAG ∆*flgBC*6143::*tetRA* | this study |
| EM6223 | *fliP*22354::G_157_-3×FLAG ∆*fliQ*22965 ∆*flgBC*6143::*tetRA* | this study |
| EM6224 | *fliP*22354::G_157_-3×FLAG ∆*fliR*22967 ∆*flgBC*6143::*tetRA* | this study |
| EM6228 | *fliO*::3×FLAG *fliP*::Q_22_-3×HA *fliR*22356::3×FLAG DydiV::FKF ∆*flgBC*6143::*tetRA* | this study |
| EM6229 | *fliO*::3×FLAG *fliP*22354::G_157_-3×FLAG *fliR*22357::TwinStrep ∆*flgBC*6143::*tetRA* ∆*ydiV*::FKF | this study |
| EM6254 | *fliO*22315::HaloTag ∆*fliF*5629::FKF | this study |
| EM6256 | *fliO*22315::HaloTag ∆*flhBAE*7669::FKF | this study |
| EM6258 | *fliN*22319::HaloTag ∆*flhBAE*7669::FKF | this study |
| EM6394 | *lamB*::T_26_-*lacZ*-FKF / pTrc99AFF4-STM1085_∆aa5-12_ | this study |
| EM6396 | *lamB*::T_181_-*lacZ*-FKF / pTrc99AFF4-STM1085_∆aa5-12_ | this study |
| EM6397 | *lamB*::T_26_-*lacZ*-FKF / pTrc99AFF4 | this study |
| EM6399 | *lamB*::T_181_-*lacZ*-FKF / pTrc99AFF4 | this study |
|  |  |  |
|  |  |  |
| Plasmid | **Relevant genotype** | **Reference** |
| pTrc99AFF4 | IPTG-inducible expression vector | ([2](#_ENREF_2)) |
| pKG116 | NaSal-inducible expression vector | J. S. Parkinson |
| pKY070 | pTrc99AFF4-*fliO*-*fliP*_His6_ | (3) |
| pEM3191 | pTrc99AFF4-STM1085_∆aa5-12_ | this study |

**References**

1. Barlag B*, et al.* (2016) Single molecule super-resolution imaging of proteins in living *Salmonella enterica* using self-labelling enzymes. *Scientific Reports* 6:31601.

2. Ohnishi K, Fan F, Schoenhals GJ, Kihara M, & Macnab RM (1997) The FliO, FliP, FliQ, and FliR proteins of *Salmonella typhimurium*: putative components for flagellar assembly. *Journal of Bacteriology* 179(19):6092-6099.

3. Fukumura T, Makino F, Dietsche T, Kinoshita M, Kato T, Wagner S, et al. Assembly and stoichiometry of the core structure of the bacterial flagellar type III export gate complex. PLoS Biology. 2017;15(8):e2002281. https://doi.org/10.1371/journal.pbio.2002281
